# Lithic bacterial communities: ecological aspects focusing on *Tintenstrich* communities

**DOI:** 10.3389/fmicb.2024.1430059

**Published:** 2024-11-29

**Authors:** Francesca Pittino, Sabine Fink, Juliana Oliveira, Elisabeth M.-L. Janssen, Christoph Scheidegger

**Affiliations:** ^1^Biodiversity and Conservation Biology, Swiss Federal Institute for Forest, Snow and Landscape Research (WSL), Birmensdorf, Switzerland; ^2^Department of Environmental Chemistry, Swiss Federal Institute of Aquatic Science and Technology (EAWAG), Dübendorf, Switzerland; ^3^Department of Earth and Environmental Sciences, University of Milano-Bicocca, Milan, Italy

**Keywords:** Cyanobacteria, lithic bacterial communities, extreme environments, *Tintenstrich*, lichens

## Abstract

*Tintenstrich* communities (TCs) mainly comprise Cyanobacteria developing on rock substrates and forming physical structures that are strictly connected to the rock itself. Endolithic and epilithic bacterial communities are important because they contribute to nutrient release within run-off waters flowing on the rock surface. Despite TCs being ubiquitous, little information about their ecology and main characteristics is available. In this study, we characterized the bacterial communities of rock surfaces of TCs in Switzerland through Illumina sequencing. We investigated their bacterial community composition on two substrate types (siliceous rocks [SRs] and carbonate rocks [CRs]) through multivariate models. Our results show that Cyanobacteria and Proteobacteria are the predominant phyla in this environment. Bacterial *α*-diversity was higher on CRs than on SRs, and the *β*-diversity of SRs varied with changes in rock surface structure. In this study, we provide novel insights into the bacterial community composition of TCs, their differences from other lithic communities, and the effects of the rock substrate and structure.

## 1 Introduction

Bacteria colonize different substrates in extreme locations (e.g., glaciers, deserts, and deep oceans) (Swan et al., 2011; Hodson et al., 2008; Connon et al., 2007). Similarly, bare rocks are in extreme environments due to being oligotrophic, frequently desiccating and presenting high-temperature shifts. Despite lithic habitats representing the oldest terrestrial surface, knowledge about life on rock is still limited (Büdel and Friedl, 2021). Bacteria are the main colonizers of this environment, but archaea, algae, fungi, and lichens also play an important role in these communities (Gorbushina, 2007). Organisms adapt different strategies to survive in such environments, for example, the production of extracellular polymeric substances (EPSs) is a typical characteristic of both bacteria and fungi. EPSs provide protection against desiccation and, due to their adhesive properties, retain nutrients and promote biodeterioration (Gorbushina, 2007).

**Figure 1:**
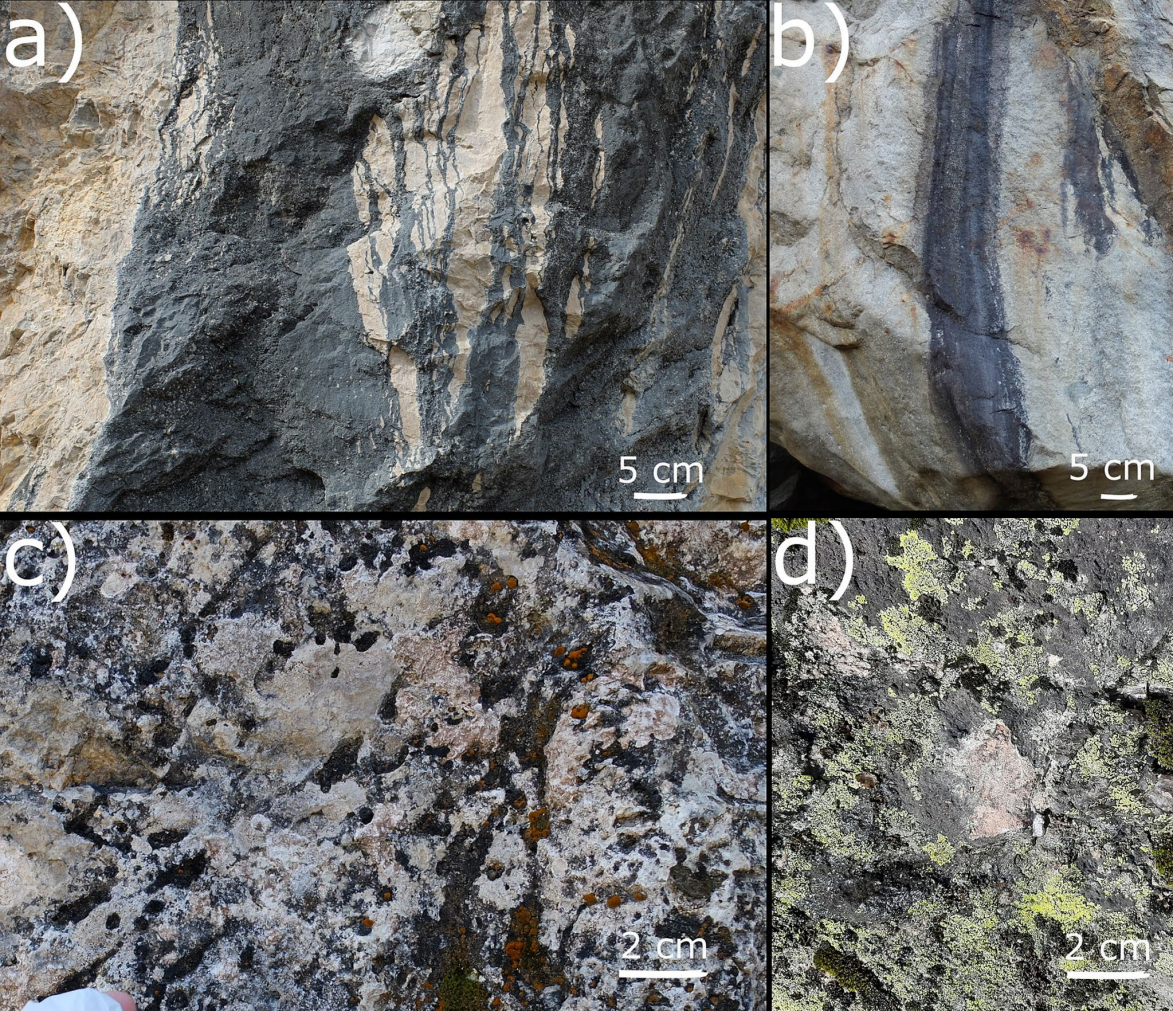
Examples of contiguous TCs on carbonate rock (CRs) (a) and siliceous rocks (b). Examples of fragmented TCs scattered among green–algal lichen communities on CRs (c) and siliceous rock (SRs) (d).

Bacteria and lichens form biofilms named *Tintenstrich* (from German “ink stripes”) communities (TCs), which are named after the dark color change of rock surface. TCs develop on rock surfaces with intermittent water runoff on different types of rock (Figures 1a,b) (Lüttge, 1997). TCs are mainly composed of free-living Cyanobacteria and cyanolichen (Büdel and Friedl, 2021; Lüttge, 1997; Porembski et al., 2000) and they cause a change in rock color, while the surface three-dimensional (3D) morphology of the rock habitat remains essentially unchanged. Typically, TCs form contiguous communities that are smooth as bare rock and become slippery when wet because of gelatinous sheaths of Cyanobacterial cells. TCs can also be fragmented among other structures, such as bryophytes and epilithic lichen associated with green algae or Cyanobacteria (Büdel and Friedl, 2021).

The main known properties promoting the development of TCs are the water retention capability of the rock (Büdel and Friedl, 2021), sun exposure (Mezzasoma et al., 2021; Lai et al., 2024), and the composition of the rock (Choe et al., 2018). TCs occur in both anthropic and natural environments: they develop on sculptures and architectural works (Gaylarde et al., 2012) and on newly exposed rocks after glaciers retreat (Haeberli et al., 2019). TCs play an important role in rock weathering and biogeochemical cycles both directly on the rock and “down valley,” where inorganic nutrients are released as a consequence of bioweathering through water flowing on the surfaces of TCs (Büdel and Friedl, 2021; Antony et al., 2012).

The relationship established between these bacterial communities and the rock substrate itself is strong. Three categories of communities are normally recognized: (i) epilithic bacteria, which colonize the external surface; (ii) hypolithic bacteria, which stay on the ventral surface of rocks (i.e., beneath pebbles); and (iii) endolithic bacteria, which develop a few millimeters deep inside the rock, where light can still penetrate and photosynthetic microorganisms can still perform photosynthesis. The endolithic communities can occupy different microhabitats inside the rock: fissures and cracks (chasmoendoliths), preexisting cavities and pores (cryptoendoliths), and actively penetrate the rock substrate (euendoliths) (Marlow et al., 2015).

These microenvironments allow the instauration of a certain variability on an apparently homogeneous rock substrate and drive different taxa to colonize different parts according to their metabolisms and characteristics (Büdel and Friedl, 2021; Khomutovska et al., 2021). Versatile metabolisms and resistance to high stresses are good characteristics that are selected for certain strains in this type of habitat. The majority of abundant bacterial phyla described in endolithic communities are Cyanobacteria, Actinobacteriota, and Proteobacteria. They colonize different types of rocks and survive different environmental conditions in aquatic and terrestrial environments (Khomutovska et al., 2021). They fulfill important ecological roles as they are involved in nutrient cycles, performing nitrogen fixation, completing the sulfur cycle, and releasing acids that decrease pH, provoking mineral dissolution from the rock (Sajjad et al., 2022). All these metabolisms are likely responsible for soil formation (Mergelov et al., 2018).

The majority of representative bacterial phylum of TCs are Cyanobacteria, a group of poikilo-tolerant microorganisms that colonize bare rock surfaces due to their metabolic properties, for example, their ability to perform photosynthesis, to fix carbon and nitrogen, to produce pigments protecting them from UV radiation and, under extreme conditions, to transform in akinetes (spore-like dormant cells) (Stanier and Cohen-Bazire, 1977). They are a photoautotrophic bacterial phylum that can colonize many different extreme environments and that can provide organic carbon and nitrogen for heterotrophic taxa (Franzetti et al., 2017).

The majority of the studies conducted on Cyanobacteria focused on toxin-producing blooms in aquatic environments (Dittmann et al., 2015), soil crusts (Beraldi-Campesi and Retallack, 2016), and on glaciers, where Cyanobacteria are regarded as pioneer colonizers (Anesio et al., 2017). However, the role of Cyanobacteria on rock surfaces, such as TCs, has been overlooked thus far. While few studies described bacterial communities on rock surfaces using both molecular and microscopic approaches, with a special focus on Cyanobacteria (Mezzasoma et al., 2021; Sajjad et al., 2022; Tang et al., 2012; Rott et al., 2021; Sigler et al., 2003), similar knowledge about TCs is not available.

The ecology of TCs was first described in 1945 by the Swiss biologists Jaag (1945) and Lüttge (1997). TCs are both epilithic and endolithic communities, but no further information exists about these important structures. In addition, other organisms (e.g., Gasteropoda) can spend part of their life on TCs, actively feeding on Cyanobacteria and cyanolichens and hence directly connecting them to the whole trophic network (Fröoberg et al., 2011).

In this article, we aim to answer three main questions:

- What are the main factors driving lithic bacterial community composition and *α*-diversity?
- Are there differences related to the rock substrate composition, light exposition, or elevation?
- Does the composition of TCs differ from other rock communities?

## 2 Materials and methods

### 2.1 Sampling design and samples collection

**Figure 2:**
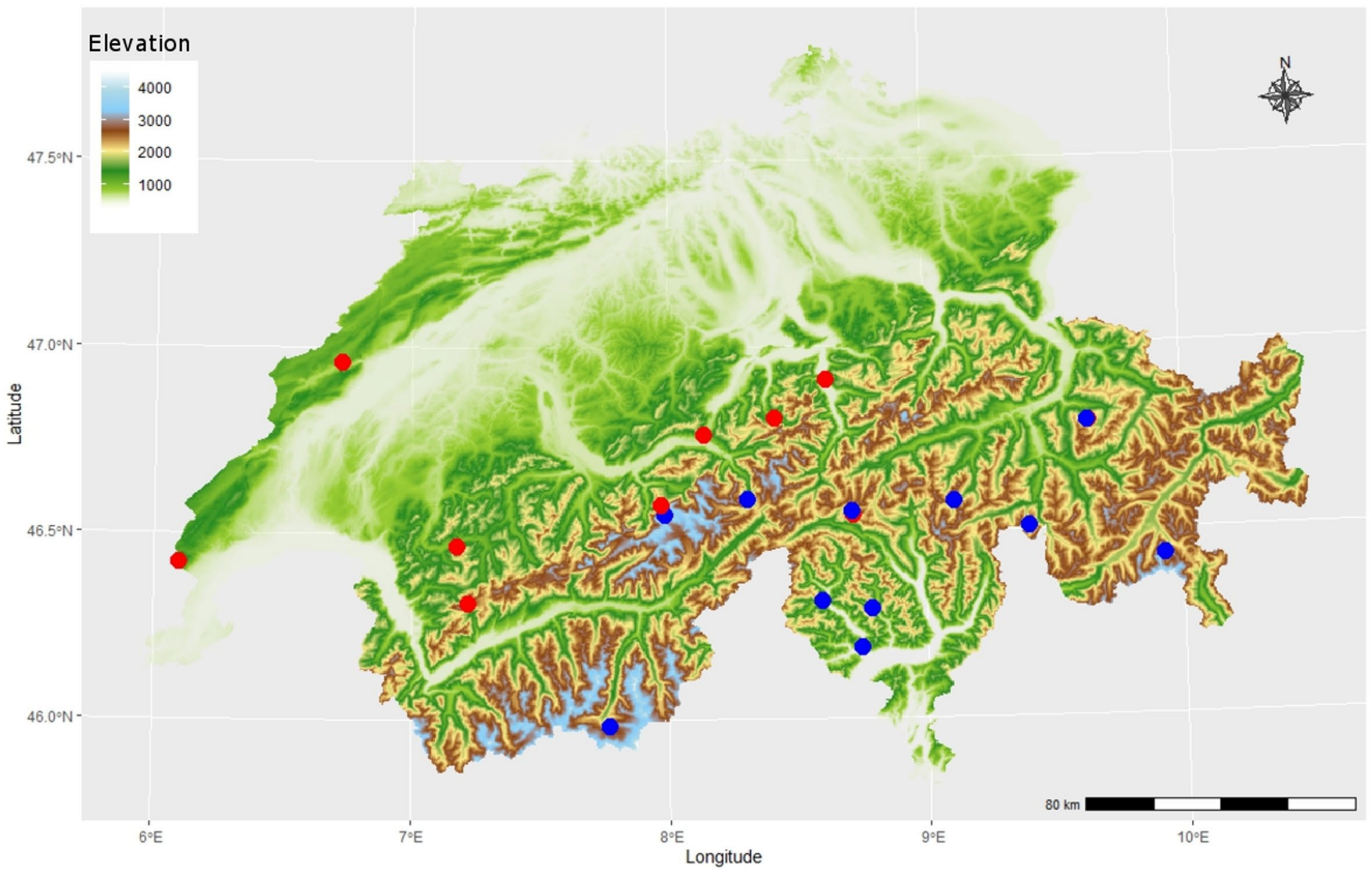
Map of 19 sampling areas in the Swiss Alpine region at different elevations (color bar), different substrates of either CRs (blue circles, *n* = 12) or siliceous rock (SRs) (red circles, *n* = 9). Replicates were taken with different light exposition (north and south facing, not shown), adding up to a total of 92 carbonate rock (CR) samples and 117 siliceous rock (SR) samples.

A total of 209 rock samples were collected in Switzerland in 19 sampling areas (Figure 2; Supplementary Table S1) with sterilized hammer and chisel. At least three replicates were collected per study site. Sampling areas were selected to obtain a similar sample number of carbonate rocks (CRs) and siliceous rocks (SRs). Some of the sampling areas were previously reported in the literature on endolithic bacteria (Sigler et al., 2003; Jaag, 1945).

Samples were collected by removing the rock with contiguous smooth TCs when present (Figures 1a,b). If contiguous TCs were unavailable, rock samples with fragmented TCs scattered among bryophytes and epilithic lichens were collected (Figures 1c,d; referred to as fragmented TC). Exposition, elevation, and fragmentation were registered. Fragmentation is used as a factor variable, which indicates how and if the rock presents classic contiguous TCs. We categorized fragmentation in detail as a three-level variable as follows: level 1 being contiguous TCs with the same 3D morphology of the rock itself and no evident growth of green–algal lichens and bryophytes (Figures 1a,b), level 2 where TCs were highly fragmented and scattered among the dominating green–algal lichens and bryophytes (Figures 1c,d), and level 3 as a condition in-between the previous two where TCs formed a macroscopically visible mosaic with bryophytes and epilithic lichens. Samples were stored at −20°C until processing.

### 2.2 DNA extraction and sequencing

DNA was extracted from rock pieces scratched from the surface (approximately from the first 3–4 mm) with the help of a sterile chisel and of a handheld rotary power tool (Dremel 8,220–1, Bosch Power Tools B.V. 2021, Netherlands). The DNA extraction was performed from 0.25 g of lyophilized scratched rock with the DNeasy PowerSoil Pro Kit (QIAGEN, Hilden, Germany), using two metal beads in a sterile 2-ml Eppendorf instead of the PowerBead Pro Tube provided in the DNeasy PowerSoil Pro Kit, and adding a 2-min vortexing in dry conditions as a first step. The other steps were performed according to the manufacturer’s instruction (protocol dated May 2019). A first polymerase chain reaction (PCR) was performed on the V4–V5 hypervariable region of the 16S ribosomal RNA (rRNA) to check for DNA quality and inhibition using the original DNA and both 1:10 and 1:100 dilutions. The PCR mixture was composed of 7.5 μL of JumpStart™ REDTaq® ReadyMix™ (Merck KGaA, Darmstadt, Germany), 0.75 μL of each primer 10 μM [515F and R926 (Sun et al., 2013)], 4.5 μL of Milliq water, and 1.5 μL of DNA. The PCR program was as follows: 3 min of initial denaturation at 95°C, 28 cycles of 45 s at 95°C, 45 s at 50°C, and 90 s at 72°C, and a final extension of 5 min at 68°C. PCR was performed using the KAPA HiFi Hotstart ReadyMix (Roche, Basel, Switzerland) and 10 μM of the two primers 515F and 806R to amplify the V4 hypervariable region of the 16S rRNA, for a final volume of 2 × 20 μL per sample. The primers were modified using Illumina adapters, a shift, and a linker, following the guidelines of Kozich et al. (2013) for 515F and Caporaso et al. (2012) for 806R. The PCR program was as follows: 3-min initial denaturation at 95°C; 28 cycles of 45 s at 95°C; 45 s at 58°C; and 45 s at 72°C, and a final extension of 5 min at 72°C. The PCR products were sent to the NGS Platform of the Institute of Genetics at Bern University (Bern, Switzerland), for sequencing with the MiSeq Illumina platform (Illumina, Inc., San Diego, CA, USA) using a 2 × 250-bp paired-end protocol.

### 2.3 Bioinformatic and statistical analyses

Demultiplexed reads were clustered in amplicon sequence variants (ASVs) with DADA2 (Callahan et al., 2016) following the pipeline reported at: https://benjjneb.github.io/dada2/tutorial.html, modifying only the truncation lengths which were of 230 and 150 for forward truncations and reverse truncations, respectively. ASVs were taxonomically classified using the SILVA database, keeping only the taxa attributed with a minimum confidence value of 0.8 (Yilmaz et al., 2014). Singletons (ASVs present in one sample only) were removed from the database to avoid inflation of the variance explained by multivariate tests (Borcard et al., 2011).

*α*-diversity was investigated through the number of ASVs, the Shannon diversity index (Shannon, 1948), the Gini inequality index (Gini, 1912), the Chao I index, and the Simpson index, which was calculated on a dataset rarefied to 4,155 sequences per sample; this number is slightly less than the lowest number of sequences in a sample.

*β*-diversity was investigated on a non-rarefaction dataset where ASV numbers were transformed with the Hellinger distance (De Cáceres et al., 2010). *β*-diversity was investigated through the Canonical Correspondence Analysis (CCA) of the ASVs’ abundances transformed with the Chi-square distance used as predictors—northness [cos(aspect)], eastness [sin(aspect)], elevation, and the interaction between rock type (CR or SR) and fragmentation.

*Post-hoc* tests (Tukey’s method) were used to assess pairwise differences in the structure of bacterial communities between rock and levels of fragmentation, correcting *p*-values with the false discovery rate (FDR) method using the Benjamini–Yekutieli procedure (Benjamini and Yekutieil, 2001). Variation partitioning was used to quantify the variation of community structures according to the same variables used in the CCA. The majority of abundant phyla and orders were also investigated through generalized linear models (GLMs) with a poisson distribution corrected for overdispersion and correcting *p*-values using the same FDR procedure as above. The majority of abundant taxa were those that, when summed up, represented more than 80% of the community’s ASVs. The multipatt R function of indecspecies was used on the majority of abundant genera to investigate indicator species in TCs, correcting *p*-values with the FDR method using the Benjamini–Yekutieli procedure. Indicator genera were investigated considering all the ecologically significant groups of the three levels of the variable level of fragmentation. We considered all the fragmentation levels as ecologically significant in combination with the rock type, fitting them along a fragmentation gradient.

Shared ASVs were investigated to describe the bacterial communities in samples collected from contiguous TCs of CRs and SRs, merging those from the same rock wall. Analyses were performed using R 4.2.1 (R Core Team, 2022) with the VEGAN, BIODIVERSITYR, MULTTEST, INDICSPECIES, and MULTCOMP packages. Shared ASVs among contiguous CR TC samples and among contiguous SR TC samples were investigated following the conservative assumption that they strictly represent TCs, providing data about the core community of these structures without too much noise. ASVs resulting in interesting indicator genera were blasted on the National Center for Biotechnology Information (NCBI) database.^1^

## 3 Results

We collected 92 CR samples and 117 SR samples from a total of 19 sampling areas. The CR samples had an elevation range of 500–2,930 m a.s.l. with a median of 2,000 m a.s.l., of which 35 samples were from contiguous TCs, 30 samples were mainly fragmented rocks, and 27 samples were both contiguous TCs and fragmented rock surface. SR samples had an elevation range of 370–3,500 m a.s.l. with a median of 2,105 m a.s.l., of which 48 samples were contiguous TCs, 40 samples were mainly fragmented rocks and 29 samples were both contiguous TCs and fragmented rock surface.

**Figure 3:**
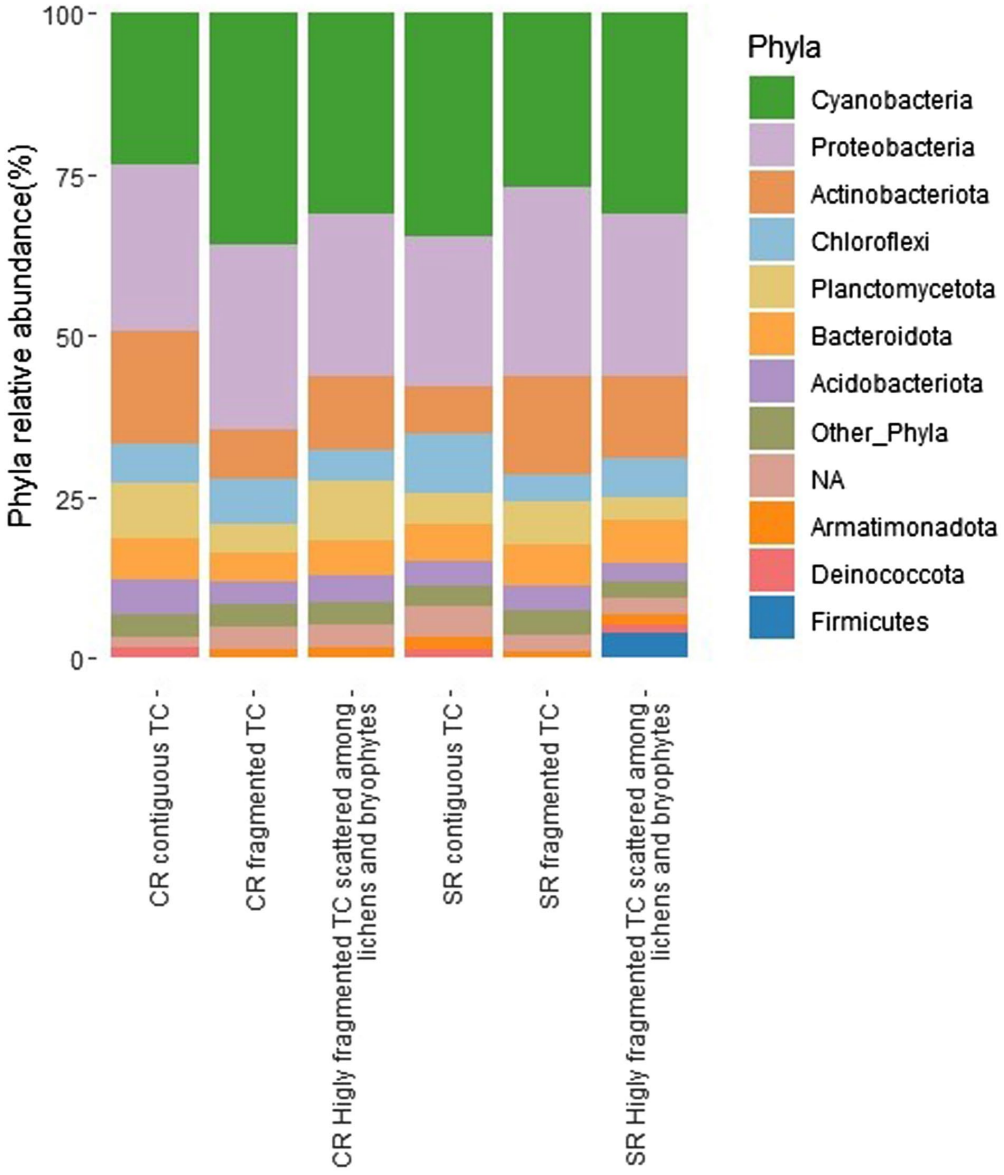
Relative abundance of bacterial phyla expressed as the percentage of sequences for the different conditions (fragmentation and type of rock, CRs, carbonatic rocks; SRs, siliceous rocks; TCs, *Tintenstrich* communities). Phyla, whose abundance was <1%, were grouped in “Other_Phyla”.

The number of sequences per sample ranged between 4,156 and 181,806. The majority of abundant phyla in descending order were Cyanobacteria, Proteobacteria, Actinobacteriota, Chloroflexi, and Planctomycetota (Figure 3).

At the order level, the majority of abundant orders, listed in descending relative abundance, were Cyanobacteriales, Acetobacterales, Sphingomonadales, Solirubrobacterales, Rhizobiales, Isosphaerales, Cytophagales, Thermomicrobiales, Gemmatales, Blastocatellales, Ktedonobacterales, Leptolyngbyales, Burkholderiales, Caulobacterales, Rhodobacterales, Rubrobacterales, Frankiales, Pseudomonadales, Propionibacteriales, Pseudonocardiales, Chitinophagales, Armatimonadales, Deinococcales, Pyrinomonadales, Tistrellales, Tepidisphaerales, Gemmatimonadales, Chthoniobacterales, Gaiellales, Chloroflexales, Kallotenuales, Abditibacteriales, Gloeobacterales, Microtrichales, Bacillales, Thermosynechococcales, Vicinamibacterales, uzebyales, Micrococcales and Kineosporiales (Supplementary Figure S1).

**Figure 4:**
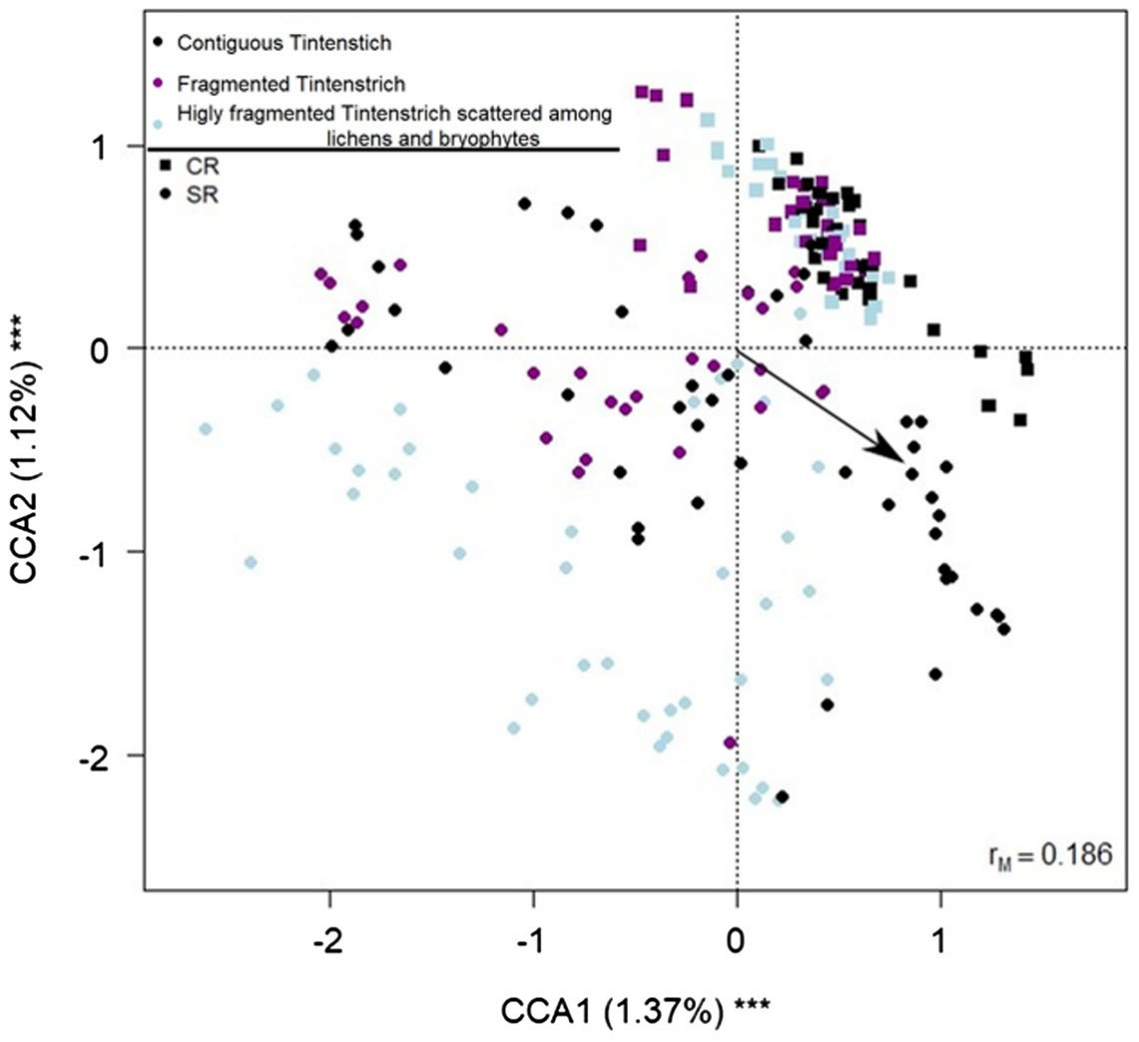
Biplot from the canonical correspondence analysis (CCA) on bacterial amplicon sequence variants (ASV) abundance on fragmentation, rock type, elevation, northness, and eastness. Each data point represents one sample. The fragmentation is indicated by different colors (black = contiguous TCs, purple = both contiguous TCs and fragmented surface, light blue = fragmented rock surface). The arrow indicates the increase in elevation. Squares indicate CR samples, and circles SR samples. The percentage of variance explained by each axis and its significance (***p < 0.001) is reported. rM is the Mantel correlation coefficient between the Chi-square distance between samples and the Euclidean distance between the corresponding symbols in the graph. Values close to one indicate that the graph correctly represents the distance between samples.

The CCA showed that all the variables included in the model were significant in explaining the variance of the bacterial community (Table 1; Figure 4; see also Supplementary Figure S2, which shows the same plot as Figure 4 with a focus on selected sampling sites (Piora Valley and Weisshorn) where both CRs and SRs were found).

**Table 1:** Canonical correspondence analysis (CCA) of variance of bacterial amplicon sequence variants (ASV) abundance according to the interaction of fragmentation and rock type (CR or SR), elevation, northness, and eastness.

Variable	*df*	Variance	*F*	*p*
Fragmentation and rock type	2	0.569	1.289	0.001
Elevation	1	0.585	2.652	0.001
Eastness	1	0.280	1.270	0.002
Northness	1	0.330	1.496	0.001
Residuals	200	44.577		

F_8,200_ = 1.661, *p* = 0.001, *R*^2^_adj_ = 0.025.

The variation partitioning revealed that the interaction between the type of rock and the fragmentation explained the majority of the variance (0.2%), also the elevation explained part of the variance (0.1%), while eastness and northness did not explain a part of the variance significantly different from zero. *Post-hoc* tests on the interaction between rock and fragmentation revealed that the bacterial community changed significantly among all the comparisons (|t_200_| ≥ 1.208, P_FDR_ ≤ 0.046) except between contiguous CRs and contiguous CR with fragmented rock surface (|t_200_| = 0.949, P_FDR_ = 1).

**Figure 5:**
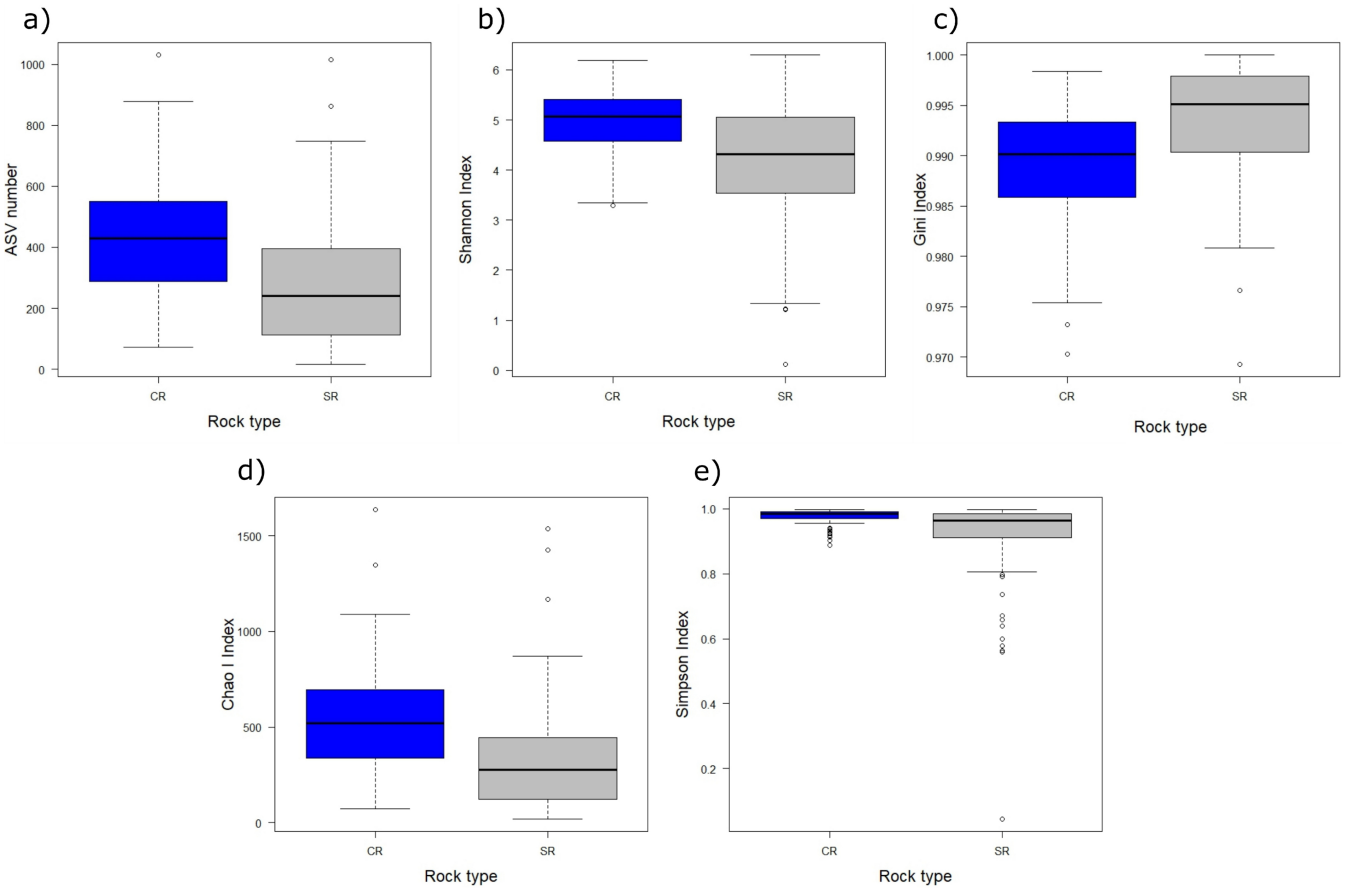
Boxplots of the amplicon sequence variants (ASV) number (a), Shannon index (b), Gini index (c), Chao I index (d), and Simpson index (e) in relation to the rock substrate (blue = CRs, gray = SRs). The thick lines represent the median, boxes upper and lower limits the 25th and the 75th percentiles, respectively, whiskers the data points beyond the 5th percentile (lower whisker) and the 95th percentile (upper whisker), and open circles represent the outliers.

The analyses on the *α*-diversity showed that the number of ASVs, Gini index, Shannon index, Simpson index, and Chao I index did not change with the elevation (F_8,200_ ≥ 3.402, P_FDR_ ≥ 0.0636), eastness (F_8,200_ ≥ 0.0272, P_FDR_ = 1), northness (F_8,200_ ≥ 0. 01, P_FDR_ ≥ 0.473), fragmentation level (F_8,200_ ≥ 0.204, P_FDR_ ≥ 0.083), and the interaction between rock and fragmentation (F_8,200_ ≥ 0.176, P_FDR_ ≥ 0.151). Significant differences were detected only among rock types (F_8,200_ ≥ 13.613, P_FDR_ < 0.001) for all indices, ASV number, Shannon index, Simpson index, and Chao I index were higher in CR samples than in SRs. In contrast, the Gini index was lower in CRs (Figure 5).

The GLM results of the majority of abundant phyla and orders showed that only Actinobacteriota (F_2,200_ = 7.214, P_FDR_ = 0.0108) (Supplementary Figure S3e) and their order Solirubrobacterales (F_2,202_ = 8.481, P_FDR_ = 0.0183) (Supplementary Figure S4) changed with the interaction between fragmentation and rock type. No phylum changed according to fragmentation (F_2,200_ ≥ 0.179, P_FDR_ ≥ 0.859). Still, at the order level, Cyanobacteriales changed (F_2,202_ = 6.871, P_FDR_ = 0.019) (Supplementary Figure S5), resulting in more abundant in contiguous TCs and less abundant in the presence of predominantly fragmented rock.

Cyanobacteria, Actinobacteriota, Chloroflexi, and Planctomycetota changed with the rock substrate (F_1,200_ ≥ 8.666, P_FDR_ ≤ 0.0103) (Supplementary Figures S3a–d). At order level Acetobacterales, Sphingomonadales, Solirubrobacterales, Rhizobiales, Isosphaerales, Thermomicrobiales, Gemmatales, Ktedonobacterales, Leptolyngbyales, Burkholderiales, Caulobacterales, Rhodobacterales and Rubrobacterales changed according to rock type (F_1,202_ ≥ 8.002, P_FDR_ ≤ 0.0231) (Supplementary Figure S6). In particular, on SRs, there were more Cyanobacteria and Chloroflexi and at order level Leptolyngbyales and Ktedonobacterales (belonging to Cyanobacteria and Chloroflexi phyla, respectively) and Acetobacterales belonging to the phylum Proteobacteria. On CRs instead, there were more Actinobacteriota and two orders belonging to this phylum (i.e., Solirubrobacterales and Rubrobacterales), Planctomycetota and two orders belonging to this phylum (i.e., Isosphaerales and Gemmatales), five orders belonging to the phylum Proteobacteria (i.e., Rhizobiales, Sphingomonadales, Burkholderiales, Caulobacterales, and Rhodobacterales), Cytophagales belonging to the phylum Bacteroidota, and finally the order Thermomicrobiales belonging to the phylum Chloroflexi.

The majority of abundant phyla changed with elevation (F_1,200_ ≥ 5.549, P_FDR_ ≤ 0.0444) (Supplementary Figure S7), and also seven of the majority of abundant orders (namely, Cyanobacteriales, Solirubrobacterales, Rhizobiales, Gemmatales, Ktedonobacterales, Burkholderiales, and Frankiales) changed with elevation (F_2,202_ ≥ 11.482, P_FDR_ ≥ 0.00761) (Supplementary Figure S8). In particular, a decrease with elevation resulted in the following: Cyanobacteria and their order Cyanobacteriales, Chloroflexi and their order Ktedonobacterales, and Planctomycetota and their order Gemmatales and finally the order Rhizobiales. On the contrary, Proteobacteria and their order Burkholderiales and Actinobacteriota and their orders Solirubrobacterales and Frankiales increased with higher elevation.

Proteobacteria (F_1,200_ = 9.610, P_FDR_ = 0.0253) (Supplementary Figure S9) and their order Burkholderiales (F_1,202_ = 16.76, P_FDR_ = 0.00387) were the only taxa changing with eastness.

Plantomycetota was the only phylum that changed with northness (F_1,200_ = 8.935, P_FDR_ = 0.0359) (Supplementary Figure S9). At order level, Isosphaerales belonging to Plantomycetota, Acetobacterales and Rhizobiales belonging to the phylum Proteobacteria, and Ktedonobacterales belonging to the phylum Chloroflexi also exhibited changes with northness (F_1,202_ ≥ 9.442, P_FDR_ ≤ 0.038) (Supplementary Figure S10). Indicator species analyses revealed that on CRs, no indicator genera were identified in areas where both contiguous TCs and fragmented rock surfaces were present, nor when combining data from contiguous TCs alone and fragmented rocks. On the other hand, on SRs, no indicator genera were detected in the fragmented rock areas (Table 2). All the seven ASVs classified as the genus *YB-42* were subsequently blasted on the NCBI, and they all had the best match with the strain *Chroakolemma pellucida 719*.

**Table 2:** Results of analysis for indicator genera for different levels of fragmentation and/or different rock substrates and their ecologically relevant combinations for TCs.

Fragmentation	Rock	
	CR	SR
Contiguous TCs	*Paracoccus, Lawsonella, Dolosigranulum*	*Streptococcus, Tepidimonas, Cohnella*
Both contiguous TCs and fragmented rock surface	n.d.	*JG30a-KF-32*
Contiguous TCs and both contiguous TCs and fragmented rock surface	n.d.	*YB*-42
Fragmented rock surface	n.d.	n.d.
Fragmented rock surface and both contiguous TCs and fragmented rock surface	n.d.	*Bradyrhizobium, Bryocella, Acidisoma, Acidicaldus*
All levels	*Rubellimicrobium, PMMR1*, *Oceanicella*, *MIZ36*, *Lysobacter, OM27 clade*	*Rubritepida*
All rock samples are contiguous TCs	*Acinetobacter, Sporosarcina, Haemophilus*	
All rock samples contiguous TCs and both contiguous TCs with fragmented rock surface	*Herpetosiphon, Stenotrophomonas*	
All samples	*Acidiphilium, Sphingomonas, Spirosoma, Aliterella, Fimbriiglobus, Solirubrobacter, Rubrobacter, Blastocatella, Pseudonocardia, Synechococcus PCC*-*7502*, *Aurantisolimonas, Rhizobacter, Kribbella, Chlorogloea SAG*-*10*-*99*, *Allorhizobium-Neorhizobium-Pararhizobium-Rhizobium*	

n.d., indicator genera were not detected.

Finally, we evaluated the shared bacterial ASVs in contiguous TC samples, dividing these samples into two big groups, CRs and SRs. The results showed that 39 ASVs were shared among contiguous CR TC samples. At the genus level, 20 of these ASVs were unclassified, while the others included *Acidiphilium*, *Psychroglaciecola, PMMR1, Candidatus Udaeobacter, Fimbriiglobus, Hymenobacter, Pseudonocardia, Rubrivirga, Solirubrobacter, Sphingomonas*, and *Truepera* (Supplementary Table S2). In SRs, no shared ASVs were detected among contiguous TCs.

## 4 Discussion

This study describes the lithic bacterial communities of alpine areas of Switzerland, with a novel focus on TCs using a metabarcoding approach. Overall, our results show that bacterial communities vary according to rock substrate, elevation, and exposition and TCs differ depending on the rock substrates.

### 4.1 TCs host unique communities compared to other rock morphologies

Our results show that Cyanobacteria and Proteobacteria are the predominant phyla of rock bacterial communities. This is consistent with the current knowledge of bacterial lithic communities and TCs (Lüttge, 1997; Jaag, 1945; Louati et al., 2020; Porembski et al., 2000). The only exceptions were reported for sandstone in Antarctica, where Proteobacteria and Actinobacteriota were the majority of predominant phyla, while Cyanobacteria were under-represented (Coleine et al., 2019). A second study reported communities with only one Cyanobacterial phylotype on sandstone (Mezzasoma et al., 2021; Coleine et al., 2019). Cyanobacteria were also under-represented (Choe et al., 2018) in travertine samples of the Arctic, and the authors suggest that this may be the effect of the rock’s chemical composition. Our data can neither confirm nor reject these results because we excluded sandstone in the sampling design and no travertine is present in our sampling region (Geologischer Atlas Der Schweiz 1:25000, 1930).

TCs hosted different communities than other rock morphologies, and exposition, elevation, fragmentation, and rock substrate contributed to the variation of the communities. These variables are intrinsically related to water availability on the rock, known to affect the microbial community. The samples within Weisshorn and Piora Valley areas including both rock types, clustered separately along the *y*-axis of the CCA, suggesting that the rock substrate can have a stronger effect than geographic location. This is consistent with previous results (Brewer and Fierer, 2018) showing that differences in the tomb’s communities changed according to the geographic area and the substrate (limestone or granite). The CCA analyses show that the variance explained by the axes is low but significant, probably due to the high within-sample variability. Similarly, it has already been reported that microenvironmental characteristics drive the community composition (e.g., rock microarchitecture) (Khomutovska et al., 2021).

### 4.2 TCs on CRs show a higher diversity

Regarding *α*-diversity, the results showed that both richness and evenness are higher on CRs than on SRs. This result is consistent with the fact that the nutrients in CRs are more limited to calcium carbonate (Kinzel, 1983), leading subsequently to lower competition as the result of a more oligotrophic environment, and a similar result was already reported in Svalbard lithic communities (Choe et al., 2018). Furthermore, CRs are more subjected to bioerosion and rock weathering, and therefore they are a more ephemeral substrate where this dynamicity may obstruct the instauration of a few specific bacterial populations that need more time to develop (Büdel and Friedl, 2021; Lerman and Meybeck, 1988). The respiration of endolithic lichens and all the other microorganisms inhabiting the rock substrate increases CO_2_ concentrations forming H_2_CO_3_ that subsequently decreases pH and promotes ion leaching (Gorbushina, 2007). As CR is a particularly dense substrate, bioweathering is also a survival strategy adopted by the communities to colonize the inner layers and avoid the epilithic environment where different stressors are stronger (e.g., UV radiation) (Weber et al., 2011). Bioweathering is caused by only a subset of the total community, but other taxa can still take advantage of it. Contrary to CRs, SRs are more resistant to rock weathering. Different materials of both mineral and biological origin can consequently accumulate on SR surfaces, forming microlayers that can act as traps where nutrients can accumulate. Indeed, these microlayers often present EPS that have adhesive properties keeping the microorganisms together, promoting their inclusion in the substrates and also the retention of water and nutrients (Gorbushina, 2007). Finally, CR is also more porous and this can lead to higher water retention, promoting the development of different bacterial populations (Chen et al., 2023). No differences in α-diversity indices were detected according to exposition or elevation.

### 4.3 Unstable (CR) and stable (SR) rock environments host different taxa

GLMs revealed that Actinobacteriota changed according to the interaction between fragmentation and rock substrate. Their relative abundance was constant on CRs, while there was a reduction related to decreased fragmentation on SRs. Actinobacteriota are a ubiquitous phylum colonizing extreme environments and have already been reported to occur in the lithic substrate (Coleine et al., 2019; Zhang et al., 2014). Analogously, their order Solirubrobacterales also changed with the interaction between rock type and fragmentation, but not much information is available to explain this trend.

Changes in fragmentation were observed in TCs. In particular, on contiguous TC, the order Cyanobacteriales had a higher relative abundance, confirming that Cyanobacteria are enriched on TCs. This order provides information about filamentous and non-filamentous Cyanobacteria (e.g., *Nostoc* and *Gloeocapsa*) and is coherent with previous results (Mihajlovski et al., 2017). When examining the cyanobacterial sequences in contiguous TCs alone, the majority (60.74%) were classified at the family level as Chroococcidiopsaceae, followed by Nostocaceae (11.71% of the sequences). These observations are consistent with the fact that both filamentous and non-filamentous Cyanobacteria are involved in TC formation, with a predominance of non-filamentous ones. This aspect is not surprising since, even if the majority of the known cyanobionts are filamentous, there is evidence that unicellular cyanobionts exist and may be underestimated due to technical difficulties in their study (Jung et al., 2024). Furthermore, there is no match with our results of the indicator genera analysis, where no genera belonging to this order are reported for all contiguous TC samples. The lack of indicator genera may suggest more differentiation according to the rock type and other environmental variables at the lowest taxonomic levels.

Changes in taxa composition with rock substrate type were also observed. At the phylum level, Actinobacteriota and Planctomycetota were more abundant on CRs, while Cyanobacteria and Chloroflexi were more abundant on SRs. Actinobacteriota decrease in relative abundance on SRs and especially on fragmented rock surfaces, which can be the effect of the selective pressure due to the more stable environment (with SRs being a more stable substrate than CRs, and similarly fragmented surfaces on SR are more stabe than contiguous). Overall, 50.74% of the ASVs assigned to Actinobacteriota were unclassified. Among the classified genera belonging to this phylum, which were 71, the majority of abundant were *Solirubrobacter* (10.2%), *Rubrobacter* (10.1%), and *Pseudonocardia* (7%). All the other genera were less than 3% of all Actinobacteriota ASVs. Actinobacteriota are known to colonize rocks and can become dominant, especially on volcanic rocks (Cockell et al., 2013). As Cockell et al. (2013) emphasize, much information is still lacking at the lowest taxonomic levels due to the limits of culturing this phylum. Similar trends to Actinobacteriota were found for Planctomycetota, which are ubiquitous phyla inhabiting extreme environments.

The higher relative abundances of Cyanobacteria and Chloroflexi on SRs have different possible causes. First, Cyanobacteria are not only present as free-living bacteria, but also as cyanolichen, and our molecular approach cannot distinguish between these two groups. When scratching the rock samples, it was not possible to differentiate the biomass obtained from the rock between lichen and non-lichen material, partly because lichens can also grow as endoliths (Wong et al., 2010). Therefore, DNA was extracted from the whole rock surface. A similar result was observed by Purahong et al. (2021), who showed that Cyanobacteria diversity was negatively affected in highly disturbed areas. Chloroflexi are known to be involved in the formation of matrixes that allow the aggregation of external material, making them more likely on a more stable substrate (Speirs et al., 2019).

Differences with the rock substrate were observed at the order level as well. First, for the order Acetobacterales, the majority of the sequences of this order were classified as *Acidiphilium* at the genus level (48.8%). It was the far more abundant genus within this order (43.5% of the sequences were unclassified). The frequent classification of *Acidiphilium* is consistent with its higher relative abundance on SRs, a more acidic substrate, and, therefore, a more suitable environment for this genus (Rosenberg, 2013).

The abundance of Rhizobiales changed according to the rock substrate type, despite 58% of their sequences being unclassified. Their higher relative abundance on SR is consistent with the fact that SRs are a more stable environment where root systems can develop, which is known to represent a good substrate for the majority of the genera belonging to this order (Lemos et al., 2021).

The relative abundance of the order Gemmatales was higher on CRs. This order is chemoheterothrophic with an optimum growth pH typically being neutral-acidic (Dedysh, 2020). Therefore, these CR-inhabiting Gemmatales genera may be more neutrophilic to colonize this rock type better than SR. Similar to their phylum Actinobacteriota, Solirubrobacterales were more abundant on CRs. This order is known to be present in the soil, and it positively correlates with the presence of nutrients but decreases with soil erosion (Qiu et al., 2021). Therefore, our results were rather unexpected since CR is more oligotrophic and more subjected to erosion and weathering phenomena (Büdel and Friedl, 2021; Lerman and Meybeck, 1988). Nevertheless, the CR erosion rate is slower than soil one; therefore, the two substrates may not be comparable.

Ktedonobacterales were of higher abundance on SRs, while they decreased with elevation and exposition. Their predominance on SRs is consistent with a previous discovery in a quartzite cave, where Ktedonobacterales were dominant in the first stage of orthoquartzite rock alteration (Ghezzi et al., 2021). At the order level, 66.17% of their sequences were unclassified, while the majority of abundant genus was *1959–1* (32.4%).

The order Leptolyngbyales was also found to be more abundant on SRs, a result that is consistent with our analysis identifying the SILVA genus *YB-42* from this order as an indicator cyanobacterium for TCs on SRs. Previous results, on the other hand, show that the order Leptolyngbyales is also present in sandstone and dolomite (Gaylarde et al., 2012; Sajjad et al., 2022; Tang et al., 2012; Sigler et al., 2003) and is already known to be composed of rock biofilm-forming bacteria (Pecundo et al., 2023). Therefore, this order may be compatible with the rock environment, and possible substrate-specificity may occur at more specific taxonomic levels.

The order Caulobacterales was more abundant on CRs, and the majority of its sequences were classified at the genus level as *PMMR1* (77.6%). While not much information is available about this order, the presence of CR is consistent with the oligotrophic nature of Caulobacterales (Wilhelm, 2018). Rhodobacterales were more abundant on CR, and 82% of the sequences were classified at the genus level as *Rubellimicrobium*. This genus is found in both air and soil and is known for its tolerance to metals (Yin et al., 2022; Rosenberg et al., 2014) and radiation. It has previously been detected as more abundant on CRs than on SRs in tombs, a trend thought to be related to its neutrophilic characteristics (Brewer and Fierer, 2018).

Finally, Rubrobacterales were more abundant in CR samples, and all sequences were classified as *Rubrobacter*. This genus can tolerate radiation and desiccation (Albuquerque et al., 2014).

### 4.4 Taxa relative abundance changes with elevation and exposition

The abundance of Cyanobacteria, Chloroflexi, and Planctomycetota decreased with elevation, while the abundance of Proteobacteria and Actinobacteriota showed the opposite trend. We suggest that this is because the environment is less stable at higher elevations in Switzerland because of more durable snow-packs and extreme conditions. Furthermore, these phyla can also form biofilms and grow hyphae, which can provide more stability on substrates (Tomczyk-Zak et al., 2013; Wink et al., 2017). Only Planctomycetota shows a different pattern from all other phyla, with a high peak in abundance at 2000 m a.s.l. (Supplementary Figure S7e). Regarding Proteobacteria and Actinobacteriota, previous results showed that they increase in light-inaccessible microhabitats (Lai et al., 2024), but such evidence did not result from our data. Conversely, the decrease of Rhizobiales with elevation may be explained by the tight relation with root systems.

Proteobacteria and Planctomycetota also showed a significant change in abundance with eastness and northness, respectively, and they both slightly decreased with higher solar expositions. The order Acetobacterales also decreased with solar exposition, possibly related to the lower persistence of water on sun-exposed surfaces and, therefore, less bioleaching and acid rock drainage (Büdel and Friedl, 2021).

Burkholderiales (64.5% of the sequences were unclassified at genus level) and Sphingomonadales (54.6% of the sequences were classified as *Sphingomonas*) were not discussed in this article because they represent heterogeneous taxonomic groups, where it is not possible to identify specific metabolisms or ecological trends (Garrity et al., 2015; Balkwill et al., 2006).

### 4.5 Indicator genera characterize different levels of fragmentation, especially on CRs

The results about indicator genera revealed differences between CRs and SRs. On CRs, contiguous TCs and all CR samples were the only two groups with indicator genera. On the contrary, SRs had indicator genera for all the levels except for fragmented rock surfaces. These results may be interpreted as a higher *β*-diversity on SR. The indicator genus of TCs on SRs (both contiguous and with fragmented rock surfaces) resulted to be *Chroakolemma pellucida 719*, a filamentous cyanobacterium first isolated in arid soils biocrust (Becerra-Absalón et al., 2018). Data about SR from the Pamir mountains revealed the presence of the family Leptolyngbyaceae (the one of *Chroakolemma pellucida 719*) in three samples out of eight, underlying that this bacterium cannot be found in all SR samples (Khomutovska et al., 2021).

All contiguous TCs of CR samples shared 20 ASVs, while no shared ASVs were detected among the samples of contiguous TCs of SR. This observation supports the hypothesis that CR has a lower β-diversity than SR. Furthermore, CR nutrients are limited to calcium carbonate; therefore, it is plausible that the total richness of the community is similar between sample sites since only genera with similar key functions and high adaptation can colonize such a nutrient-limited environment. The CCA showed that all CR samples cluster in a more restricted area (Figure 4) and the two fragmentation levels clustered similarly in CRs alone. On the contrary, a more spread distribution of the samples on SRs was found on the CCA biplot (Figure 4), and the community changed at all fragmentation levels. This spread in the samples is also consistent with the SR containing more different minerals, allowing higher community variability. More detailed information on the substrate composition may allow us to obtain more defined clusters.

Providing more data concerning these communities with a special focus on Cyanobacteria is important. Indeed, more than 2,425 secondary metabolites specifically produced by Cyanobacteria have been described, including those found in lichen symbiosis (Dittmann et al., 2015; Jones et al., 2021; Janssen et al., 2023; D’Agostino, 2023). Some of these metabolites are recognized as cyanotoxins in the water quality guidelines of the World Health Organization (2023), while many other metabolites demonstrate inhibitory effects on metabolite enzymes and other bioactivities (Janssen, 2019). While one study that investigated soil crusts did not detect cyanotoxin-producing genes (Dulić et al., 2022), their presence on the rock surface cannot be excluded, considering also the cyanobacterial biomass of endolithic and hypolithic environments has an average of 1.21 g/cm^2^ (Garcia-Pichel et al., 2003). Thus far, the majority of the studies on secondary metabolites producing Cyanobacteria have been conducted in aquatic environments. The prevalence of Cyanobacteria in TCs, emphasizes that the presence of cyanotoxin-encoding genes and other secondary metabolites from Cyanobacteria may be promising in the Swiss Alpine region.

## 5 Conclusion

This article provides the first molecular description of *Tintenstrich* bacterial communities. According to the fragmentation of TCs, these communities proved to change with the rock substrate. Cyanobacteria resulted in playing an important role in forming TCs, and *α*-diversity differences may be the result of the stability and the nutrients present in the different substrates. One cyanobacterial genus proved to be a good indicator of TCs on SRs. Still, the scarce knowledge of these environments does not allow obtaining a high resolution at the lowest taxonomic levels. More studies should focus on TCs since their ecological role and composition may be important when considered in the context of the lithic environments and the organisms interacting with them. In particular, monitoring TCs’ development and investigating both the active and the total bacterial communities may provide useful information to fully understand their dynamics.

## Data Availability

The original contributions presented in the study are publicly available in the NCBI SRA repository, under accession number PRJNA1095436. This data can be found here: https://www.ncbi.nlm.nih.gov/sra/PRJNA1095436.

## Footnotes

^1^https://www.ncbi.nlm.nih.gov/

## References

[ref1] Albuquerque L. Johnson M. M. Schumann P. Rainey F. A. Da Costa M. S. (2014). Description of two new thermophilic species of the genus Rubrobacter, Rubrobacter Calidifluminis Sp. Nov. and Rubrobacter Naiadicus Sp. Nov., and emended description of the genus Rubrobacter and the species Rubrobacter Bracarensis. Syst. Appl. Microbiol. 37, 235–243. doi: 10.1016/j.syapm.2014.03.001, PMID: 24780859

[ref2] Anesio A. M. Lutz S. Chrismas N. A. M. Benning L. G. (2017). The microbiome of glaciers and ice sheets. Npj Biofilms Microbiomes 3:1. doi: 10.1038/s41522-017-0019-0 28649411 PMC5460203

[ref3] Antony C. P. Cockell C. S. Shouche Y. S. (2012). Life in (and on) the rocks. J. Biosci. 37, 3–11. doi: 10.1007/s12038-012-9184-8 22357197

[ref4] Balkwill D. L. Fredrickson J. K. Romine M. F. (2006). “Sphingomonas and related genera” in The prokaryotes. eds. Dworkin M. Falkow S. Rosenberg E. Schleifer K. H. Stackebrandt E. (New York: Springer), 31–115.

[ref5] Becerra-Absalón I. Johansen J. R. Angeles Muñoz-Martín M. Montejano G. (2018). Chroakolemma gen. Nov. (Leptolyngbyaceae, Cyanobacteria) from soil biocrusts in the Semi-Desert central region of Mexico. Phytotaxa 367, 201–218. doi: 10.11646/phytotaxa.367.3.1

[ref6] Benjamini Y. Yekutieil D. (2001). The control of the false discovery rate in multiple testing under dependency. Ann. Stat. 29, 1165–1188. doi: 10.1214/aos/1013699998

[ref7] Beraldi-Campesi H. Retallack G. J. (2016). “Biological soil crusts: An organizing principle in drylands” in Biomedical and Life Sciences. eds. Weber B. Büdel B. Belnap J.. 226th ed (Berlin: Springer).

[ref8] Borcard D. Gillet F. Pierre L. (2011). Numerical ecology with R. New York, NY: Springer.

[ref9] Brewer T. E. Fierer N. (2018). Tales from the tomb: the microbial ecology of exposed rock surfaces. Environ. Microbiol. 20, 958–970. doi: 10.1111/1462-2920.14024 29235707

[ref10] Büdel B. Friedl T. (2021). “Life at rock surfaces” in Life in Extreme Environments. eds. Büdel B. Friedl T. (Cambridge: Cambridge University Press).

[ref11] Callahan B. J. McMurdie P. J. Rosen M. J. Han A. W. Amy J. A. Holmes S. P. (2016). DADA2: high-resolution sample inference from Illumina amplicon data. Nat. Methods 13, 581–583. doi: 10.1038/nmeth.3869, PMID: 27214047 PMC4927377

[ref12] Caporaso J. G. Lauber C. L. Walters W. A. Berg-Lyons D. Huntley J. Fierer N.. (2012). Ultra-high-throughput microbial community analysis on the Illumina HiSeq and MiSeq platforms. ISME J. 6, 1621–1624. doi: 10.1038/ismej.2012.8, PMID: 22402401 PMC3400413

[ref13] Chen J. Zhao Q. Li F. Zhao X. Wang Y. Zhang L.. (2023). Nutrient availability and acid Erosion determine the early colonization of limestone by Lithobiontic microorganisms. Front. Microbiol. 14:1194871. doi: 10.3389/fmicb.2023.1194871 37362915 PMC10289080

[ref14] Choe Y. H. Kim M. Woo J. Lee M. J. Lee J. I. Lee E. J.. (2018). Comparing rock-inhabiting microbial communities in different rock types from a high Arctic Polar Desert. FEMS Microbiol. Ecol. 94:70. doi: 10.1093/femsec/fiy070 29688499

[ref15] Cockell C. S. Kelly L. C. Marteinsson V. (2013). Actinobacteria-an ancient phylum active in volcanic rock weathering. Geomicrobiol J. 30, 706–720. doi: 10.1080/01490451.2012.758196

[ref16] Coleine C. Stajich J. E. Pombubpa N. Zucconi L. Onofri S. Canini F.. (2019). Altitude and fungal diversity influence the structure of Antarctic Cryptoendolithic Bacteria communities. Environ. Microbiol. Rep. 11, 718–726. doi: 10.1111/1758-2229.12788, PMID: 31393667 PMC8057506

[ref17] Connon S. A. Lester E. D. Shafaat H. S. Obenhuber D. C. Ponce A. (2007). Bacterial diversity in Hyperarid Atacama Desert soils. J. Geophys. Res. Biogeo. 112, 1–9. doi: 10.1029/2006JG000311

[ref18] D’Agostino P. M. (2023). Highlights of biosynthetic enzymes and natural products from symbiotic Cyanobacteria. Nat. Prod. Rep. 40, 1701–1717. doi: 10.1039/d3np00011g 37233731

[ref19] De Cáceres M. Legendre P. Moretti M. (2010). Improving Indicator species analysis by combining groups of sites. Oikos 119, 1674–1684. doi: 10.1111/j.1600-0706.2010.18334.x

[ref20] Dedysh S. N. (2020). “Gemmatales” in Bergey’s Manual of Systematics of Archaea and Bacteria, vol. 67 (Hoboken, NJ: Wiley), 1–2.

[ref21] Dittmann E. Gugger M. Sivonen K. Fewer D. P. (2015). Natural product biosynthetic diversity and comparative genomics of the Cyanobacteria. Trends Microbiol. 23, 642–652. doi: 10.1016/j.tim.2015.07.008, PMID: 26433696

[ref22] Dulić T. Svirčev Z. Malešević T. P. Faassen E. J. Savela H. Hao Q.. (2022). Assessment of common cyanotoxins in Cyanobacteria of biological loess crusts. Toxins 14, 1–16. doi: 10.3390/toxins14030215, PMID: 35324712 PMC8953721

[ref24] Franzetti A. Navarra F. Tagliaferri I. Gandolfi I. Bestetti G. Minora U.. (2017). Temporal variability of bacterial communities in Cryoconite on an alpine glacier. Environ. Microbiol. Rep. 9, 71–78. doi: 10.1111/1758-2229.12499 27897429

[ref25] Fröoberg L. Stoll P. Baur A. Baur B. (2011). Snail herbivory decreases cyanobacterial abundance and lichen diversity along cracks of limestone pavements. Ecosphere 2:art38. doi: 10.1890/ES10-00197.1

[ref26] Garcia-Pichel F. Belnap J. Neuer S. Schanz F. (2003). Estimates of global cyanobacterial biomass and its distribution. Algol. Stud. 109, 213–227. doi: 10.1127/1864-1318/2003/0109-0213

[ref27] Garrity G. M. Bell J. A. Lilburn T. (2015). “Burkholderiales Ord. Nov” in Bergey’s Manual of Systematics of Archaea and Bacteria (Hoboken, NJ: Wiley), 1.

[ref28] Gaylarde C. C. Gaylarde P. M. Neilan B. A. (2012). Endolithic phototrophs in built and natural stone. Curr. Microbiol. 65, 183–188. doi: 10.1007/s00284-012-0123-6, PMID: 22614098

[ref29] Geologischer Atlas Der Schweiz 1:25000. (1930). *Bundesamt für Landestopografie swisstopo*.

[ref30] Ghezzi D. Sauro F. Columbu A. Carbone C. Hong P. Y. Vergara F.. (2021). Transition from unclassified Ktedonobacterales to Actinobacteria during amorphous silica precipitation in a quartzite cave environment. Sci. Rep. 11, 1–18. doi: 10.1038/s41598-021-83416-5, PMID: 33594175 PMC7887251

[ref31] Gini C. (1912). Variabilità e mutabilità. Memorie di metodologica statistica. (1912) C. Cuppini.

[ref32] Gorbushina A. A. (2007). Life on the rocks. Environ. Microbiol. 9, 1613–1631. doi: 10.1111/j.1462-2920.2007.01301.x 17564597

[ref33] Haeberli W. Oerlemans J. Zemp M. (2019). The future of alpine glaciers and beyond. Oxford Research Encyclopedia of Climate Science. Oxford: Oxford University Press.

[ref34] Hodson A. Anesio A. M. Tranter M. Fountain A. Osborn M. Priscu J.. (2008). Concepts & Synthesis Emphasizing new Ideas to Stimulate Research in ecology glacial ecosystems. Ecol. Monogr. 78, 41–67. doi: 10.1890/07-0187.1

[ref35] Jaag O. (1945). Untersuchungen über die Vegetation und Biologie der Algen des nackten Gesteins in den Alpen, im Jura und im Schweizerischen Mittelland. Beiträge zur Kryptogamenflora der Schweiz. 9, 1–560.

[ref36] Janssen E. M. L. (2019). Cyanobacterial peptides beyond microcystins – a review on co-occurrence, toxicity, and challenges for risk assessment. Water Res. 151, 488–499. doi: 10.1016/j.watres.2018.12.048, PMID: 30641464

[ref37] Janssen E. M.-L. Jones M. R. Pinto E. Dörr F. Torres M. A. Rios Jacinavicius F.. (2023). S75 | CyanoMetDB | comprehensive database of secondary metabolites from Cyanobacteria (NORMAN-SLE-S75.0.2.0) [data set]. Zenodo 2023:7922070. doi: 10.5281/zenodo.7922070

[ref38] Jones M. R. Pinto E. Torres M. A. Dörr F. Mazur-Marzec H. Szubert K.. (2021). CyanoMetDB, a comprehensive public database of secondary metabolites from Cyanobacteria. Water Res. 196:117017. doi: 10.1016/j.watres.2021.117017, PMID: 33765498

[ref39] Jung P. Briegel-Williams L. Büdel B. Schultz M. Nürnberg D. J. Grube M.. (2024). The underestimated fraction: diversity, challenges and novel insights into unicellular cyanobionts of lichens. ISME Commun. 4. doi: 10.1093/ismeco/ycae069, PMID: 38966402 PMC11222712

[ref40] Khomutovska N. de los Ríos A. Jasser I. (2021). Diversity and colonization strategies of endolithic Cyanobacteria in the Cold Mountain desert of Pamir. Microorganisms 9, 1–17. doi: 10.3390/microorganisms9010006, PMID: 33375046 PMC7822004

[ref41] Kinzel H. (1983). “Influence of limestone, silicates and soil PH on vegetation” in Physiological plant ecology III. eds. Gottingen A. P. Zimmermann M. H. (New York: Springer-Verlag).

[ref42] Kozich J. J. Westcott S. L. Baxter N. T. Highlander S. K. Schloss P. D. (2013). Development of a dual-index sequencing strategy and curation pipeline for analyzing amplicon sequence data on the Miseq Illumina sequencing platform. Appl. Environ. Microbiol. 79, 5112–5120. doi: 10.1128/AEM.01043-13, PMID: 23793624 PMC3753973

[ref43] Lai Z. Liu Z. Zhao Y. Qin S. Zhang W. Lang T.. (2024). Distinct microbial communities under different rock-associated microhabitats in the Qaidam Desert. Environ. Res. 250:118462. doi: 10.1016/j.envres.2024.118462, PMID: 38367835

[ref44] Lemos L. N. Marques F. de Carvalho A. Gerber A. P. Guimarães C. Jonck C. R.. (2021). Genome-centric metagenomics reveals insights into the evolution and metabolism of a new free-living Group in Rhizobiales. BMC Microbiol. 21, 1–10. doi: 10.1186/s12866-021-02354-4, PMID: 34711170 PMC8555084

[ref45] Lerman A. Meybeck M. (1988). Physical and chemical weathering in geochemical cycles. Nucl. Phys. 13:3071.

[ref46] Louati M. Ennis N. J. Ghodhbane-Gtari F. Hezbri K. Sevigny J. L. Fahnestock M. F.. (2020). Elucidating the ecological networks in stone-dwelling microbiomes. Environ. Microbiol. 22, 1467–1480. doi: 10.1111/1462-2920.14700, PMID: 31158316

[ref47] Lüttge U. (1997). Cyanobacterial Tintenstrich communities and their ecology. Naturwissenschaften, 84, 526–534. doi: 10.1007/s001140050439

[ref48] Marlow J. Peckmann J. Orphan V. (2015). Autoendoliths a distinct type of rock-hosted microbial life. Geobiology 13, 303–307. doi: 10.1111/gbi.12131, PMID: 25879487

[ref49] Mergelov N. Mueller C. W. Prater I. Shorkunov I. Dolgikh A. Zazovskaya E.. (2018). Alteration of rocks by endolithic organisms is one of the pathways for the beginning of soils on earth. Sci. Rep. 8:3367. doi: 10.1038/s41598-018-21682-6 29463846 PMC5820250

[ref50] Mezzasoma A. Coleine C. Sannino C. Selbmann L. (2021). Endolithic bacterial diversity in lichen-dominated communities is shaped by Sun exposure in McMurdo dry valleys, Antarctica. Microb. Ecol. 83, 328–339. doi: 10.1007/s00248-021-01769-w, PMID: 34081148 PMC8891110

[ref51] Mihajlovski A. Gabarre A. Seyer D. Bousta F. Di Martino P. (2017). Bacterial diversity on rock surface of the ruined part of a French historic monument: the Chaalis Abbey. Int. Biodeterior. Biodegrad. 120, 161–169. doi: 10.1016/j.ibiod.2017.02.019

[ref52] Pecundo M. H. Chen T. Wang Y. Wen X. Hu Z. Chen H.. (2023). Stenomitos Nagquensis Sp. Nov. (Leptolyngbyaceae, Cyanobacteria) from a meadow wetland in the Tibet plateau: a novel species studied based on a Polyphasic approach. Diversity 15:536. doi: 10.3390/d15040536

[ref53] Porembski S. Becker U. Seine R. (2000). “Islands on islands: habitats on inselbergs” in Inselbergs: Biotic diversity of isolated rock outcrops in tropical and temperate regions. eds. Barthlott W. Porembski S. (Berlin: Springer), 49–67.

[ref54] Purahong W. Hossen S. Nawaz A. Sadubsarn D. Tanunchai B. Dommert S.. (2021). Life on the rocks: first insights into the microbiota of the threatened aquatic Rheophyte Hanseniella Heterophylla. Front. Plant Sci. 12, 1–16. doi: 10.3389/fpls.2021.634960 PMC8238419 34194446

[ref55] Qiu L. Zhang Q. Zhu H. Reich P. B. Banerjee S. van der Heijden M. G. A.. (2021). Erosion reduces soil microbial diversity, network complexity and multifunctionality. ISME J. 15, 2474–2489. doi: 10.1038/s41396-021-00913-1, PMID: 33712698 PMC8319411

[ref56] R Core Team (2022). R: A language and environment for statistical computing. Vienna, Austria: R Foundation for Statistical Computing.

[ref57] Rosenberg E. (2013). The prokaryotes: Alphaproteobacteria and Betaproteobacteria. Heidelberg: Springer Berlin Heidelberg.

[ref58] Rosenberg E. De Long E. F. Lory S. Stackebrandt E. Thompson F. (2014). The prokaryotes Alphaproteobacteria and Betaproteobacteria. 4th Edn. Berlin: Springer Reference.

[ref59] Rott E. Kurmayer R. Holzinger A. Sanders D. G. (2021). Contrasting endolithic habitats for Cyanobacteria in spring calcites of the European Alps. Nova Hedwigia 112, 17–48. doi: 10.1127/nova_hedwigia/2021/0615, PMID: 35282312 PMC7612483

[ref60] Sajjad W. Ilahi N. Kang S. Bahadur A. Zada S. Iqbal A. (2022). Endolithic microbes of rocks, their community, function and survival strategies. Int. Biodeterior. Biodegrad. 169:105387. doi: 10.1016/j.ibiod.2022.105387

[ref61] Shannon C. E. (1948). A mathematical theory of communication. Bell Syst. Tech. J. 27, 379–423. doi: 10.1002/j.1538-7305.1948.tb01338.x

[ref62] Sigler W. V. Bachofen R. Zeyer J. (2003). Molecular characterization of endolithic Cyanobacteria inhabiting exposed dolomite in Central Switzerland. Environ. Microbiol. 5, 618–627. doi: 10.1046/j.1462-2920.2003.00453.x, PMID: 12823194

[ref63] Speirs L. B. M. Rice D. T. F. Petrovski S. Seviour R. J. (2019). The phylogeny, biodiversity, and ecology of the Chloroflexi in activated sludge. Front. Microbiol. 10:2015. doi: 10.3389/fmicb.2019.02015 31572309 PMC6753630

[ref64] Stanier R. Y. Cohen-Bazire G. (1977). Phototrophic Prokariotes: the Cyanobacteria. Ann. Rev. Microbiol. 31, 225–274. doi: 10.1146/annurev.mi.31.100177.001301 410354

[ref65] Sun D. L. Jiang X. Wu Q. L. Zhou N. Y. (2013). Intragenomic heterogeneity of 16S RRNA genes causes overestimation of prokaryotic diversity. Appl. Environ. Microbiol. 79, 5962–5969. doi: 10.1128/AEM.01282-13, PMID: 23872556 PMC3811346

[ref66] Swan B. K. Martinez-Garcia M. Preston C. M. Sczyrba A. Woyke T. Lamy D.. (2011). Potential for Chemolithoautotrophy among ubiquitous Bacteria lineages in the Dark Ocean. Science 333, 1296–1300. doi: 10.1126/science.1203690, PMID: 21885783

[ref67] Tang Y. Lian B. Dong H. Liu D. Hou W. (2012). Endolithic bacterial communities in dolomite and limestone rocks from the Nanjiang canyon in Guizhou karst area (China). Geomicrobiol J. 29, 213–225. doi: 10.1080/01490451.2011.558560 22571668

[ref68] Tomczyk-Zak K. Kaczanowski S. Drewniak Ł. Dmoch Ł. Sklodowska A. Zielenkiewicz U. (2013). Bacteria diversity and arsenic mobilization in rock biofilm from an ancient gold and arsenic mine. Sci. Total Environ. 461-462, 330–340. doi: 10.1016/j.scitotenv.2013.04.087, PMID: 23743145

[ref69] Weber B. Scherr C. Bicker F. Friedl T. Buedel B. (2011). Respiration-induced weathering patterns of two Endolithically growing lichens. Geobiology 9, 34–43. doi: 10.1111/j.1472-4669.2010.00256.x, PMID: 20735487

[ref70] Wilhelm R. C. (2018). Following the terrestrial tracks of Caulobacter – redefining the ecology of a reputed aquatic Oligotroph. ISME J. 12, 3025–3037. doi: 10.1038/s41396-018-0257-z, PMID: 30108303 PMC6246563

[ref71] Wink J. Mohammadipanah F. Hamedi J. (2017). Biology and biotechnology of Actinobacteria. London: Springer Nature.

[ref72] Wong F. K. Y. Lau M. C. Y. Lacap D. C. Aitchison J. C. Cowan D. A. Pointing S. B. (2010). Endolithic microbial colonization of limestone in a high-altitude arid environment. Microb. Ecol. 59, 689–699. doi: 10.1007/s00248-009-9607-8, PMID: 19937324

[ref73] World Health Organization (2023). Water quality guidelines 2023. Toxic Cyanobacteria in water – a guide to their public health consequences, monitoring and management. Version 2. Geneva: World Health Organization.

[ref74] Yilmaz P. Parfrey L. W. Yarza P. Gerken J. Pruesse E. Quast C.. (2014). The SILVA and ‘all-species living tree project (LTP)’ taxonomic frameworks. Nucleic Acids Res. 42, D643–D648. doi: 10.1093/nar/gkt1209, PMID: 24293649 PMC3965112

[ref75] Yin W. Zhang B. Zhang H. Zhang D. Leiviskä T. (2022). Vertically co-distributed vanadium and microplastics drive distinct microbial community composition and assembly in soil. J. Hazard. Mater. 440:129700. doi: 10.1016/j.jhazmat.2022.129700, PMID: 35969955

[ref76] Zhang G. Cao T. Ying J. Yang Y. Ma L. (2014). Diversity and novelty of Actinobacteria in Arctic marine sediments. Antonie Van Leeuwenhoek 105, 743–754. doi: 10.1007/s10482-014-0130-7, PMID: 24519808

