# Supplementary material for: Lithic bacterial communities: ecological aspects focusing on Tintenstrich communities
Source: Front Microbiol. 2024 Nov 29;15:1430059. doi: 10.3389/fmicb.2024.1430059 (PMC11639984; doi:10.3389/fmicb.2024.1430059)
Supplement: Supplementary file 1 [file Data_Sheet_1.docx]

**Lithic bacterial communities: ecological aspects focusing on *Tintenstrich communities***

***Supplementary Material***

Francesca Pittino^1,2,3*^, Sabine Fink^1^, Juliana Oliveira^1,2^, Elisabeth M.-L. Janssen^2^, Christoph Scheidegger^1^

*^1^Biodiversity and Conservation Biology, Swiss Federal Institute for Forest, Snow and Landscape Research (WSL), Birmensdorf, Switzerland.*

*^2^Department of Environmental Chemistry, Swiss Federal Institute of Aquatic Science and Technology (EAWAG), Dübendorf, Switzerland.*

*^3^Department of Earth and Environmental Sciences, University of Milano-Bicocca, Milan, Italy*

**Corresponding Authors:* [*francesca.pittino@unimib.it*](mailto:francesca.pittino@unimib.it)

**Supplementary material**


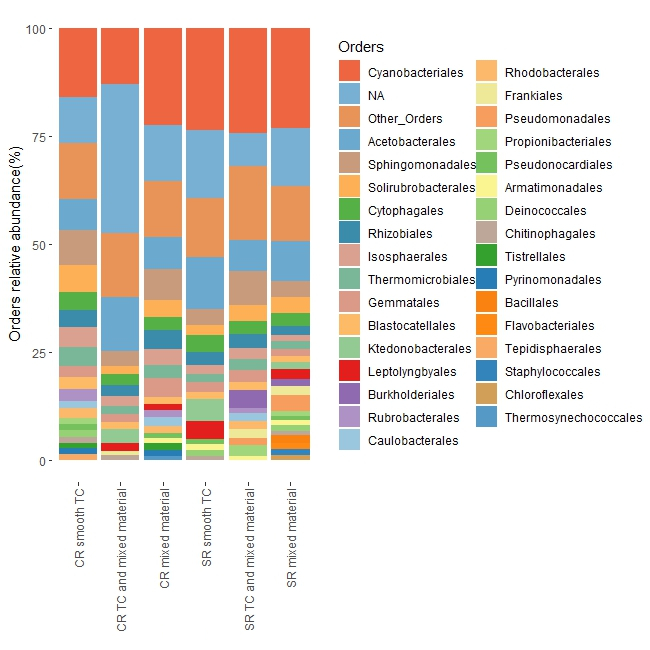


**Figure S1**. Relative abundance of bacterial orders expressed as the percentage of sequences. Only the most abundant orders are shown, those which are not included between the most abundant were grouped in ‘Other Orders’. TC indicates Tintenstrich, CR indicates carbonatic rocks and SR siliceous rocks.


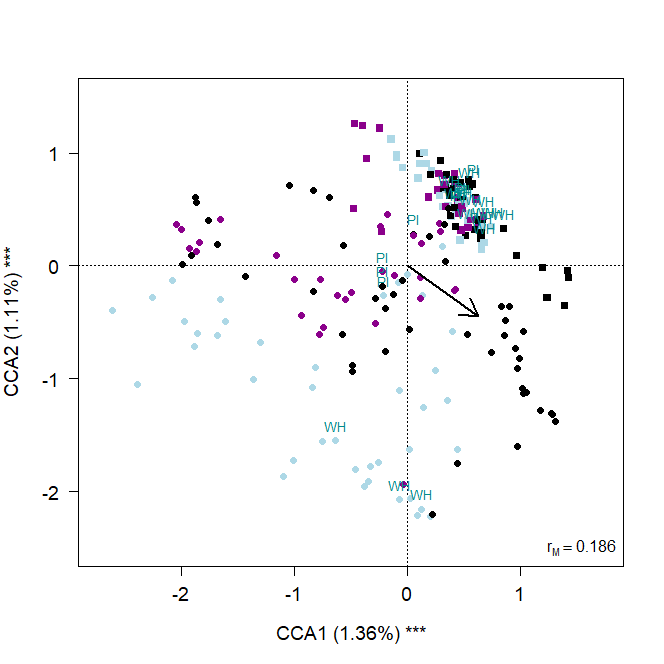


**Figure S2.** Biplot from the CCA (canonical-correlation analysis) on bacterial ASV (Amplicon Sequence Variants) abundance on smoothness, rock type, elevation, northness and eastness. Each point represents one sample. The smoothness is indicated by different colours (black = contiguous TC, purple = both contiguous TC and fragmented surface, light blue = fragmented rock surface). The arrow indicates the elevation. Squares indicate CR (carbonatic rocks) samples and circles SR (Siliceous rocks) samples. A few sampling areas are reported: WH = Wheisshorn, PI = Piora Valley. The percentage of variance explained by each axis and its significance (***: P< 0.001) is reported. rM is the Mantel correlation coefficient between the chi-square distance between samples and the Euclidean distance between the corresponding symbols in the graph. Values close to one indicate that the graph correctly represents the distance between samples.


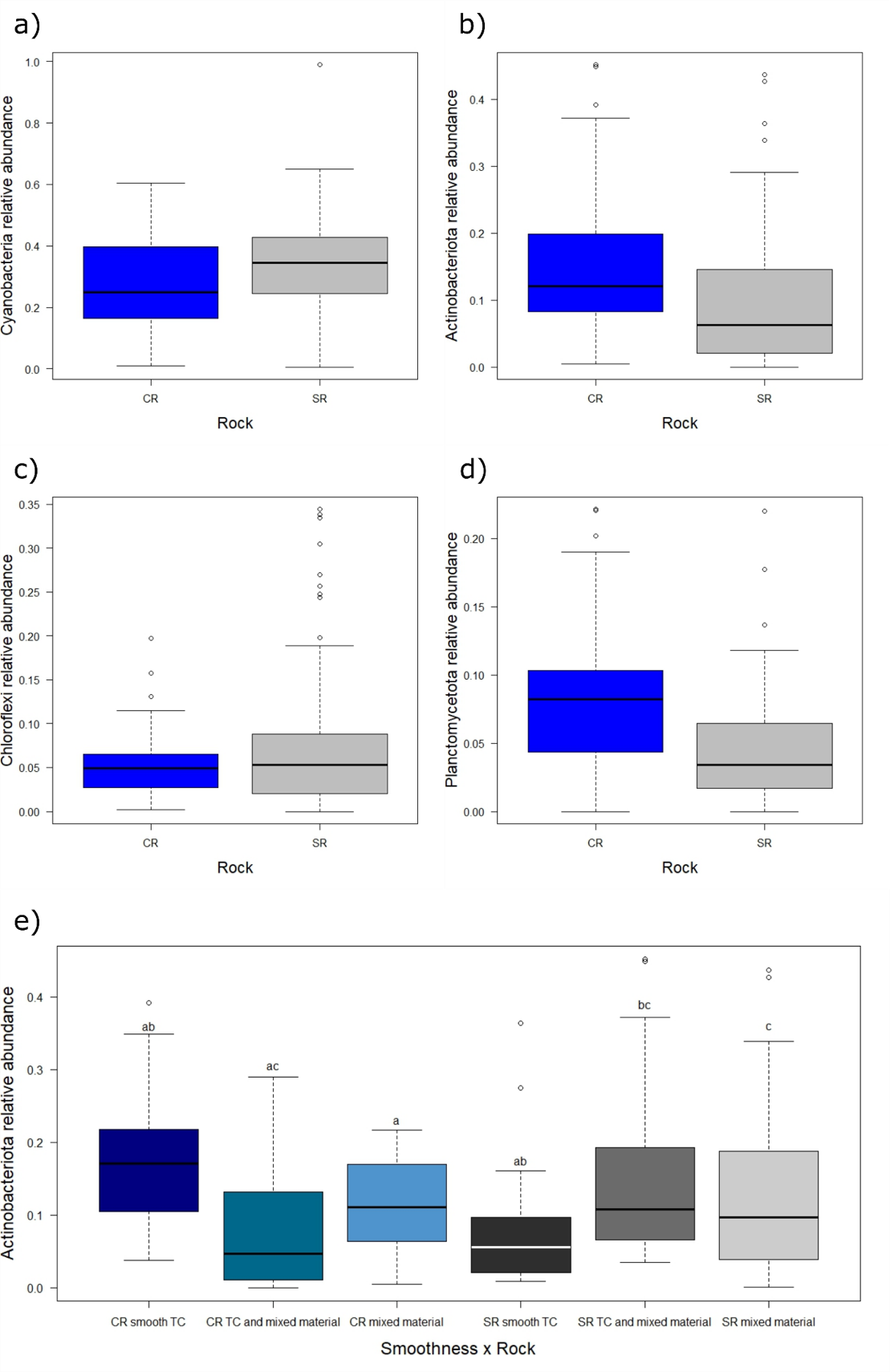


**Figure S3.** Boxplots of the relative abundances of cyanobacteria (a), actinobacteriota (b), chloroflexi (c) and planctomycetota (d) according to the rock substrates: CR (carbonatic rocks) (blue) and SR (siliceous rocks) (grey). The thick lines represent the median, boxes upper and lower limits the 25^th^ and the 75^th^ percentiles respectively, whiskers the data that go beyond the 5^th^ percentile (lower whisker) and the 95^th^ percentile (upper whisker), open circles represent the outliers and different letters indicate differences between the mean values of different groups.


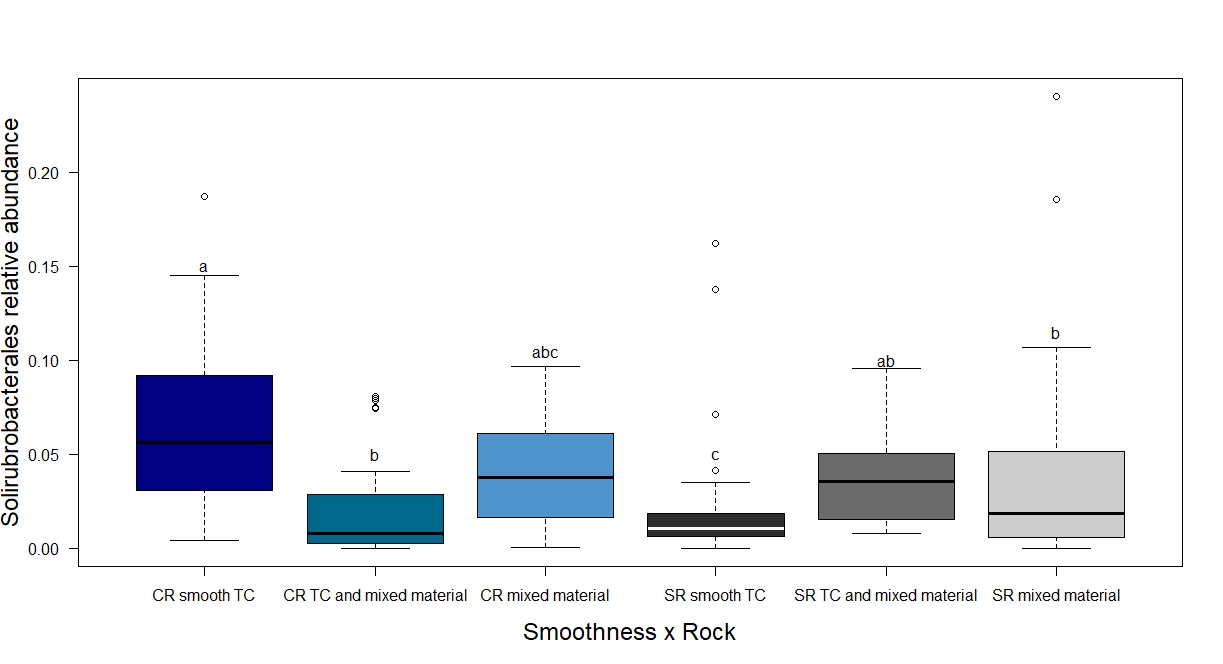


**Figure S4**. Boxplots of the relative abundances of solirubrobacterales on the interaction between smoothness and rock substrate (CR = carbonatic rocks in blue shades, SR = Siliceous Rock in grey shades). The thick lines represent the median, boxes upper and lower limits the 25^th^ and the 75^th^ percentiles respectively, whiskers the data that go beyond the 5^th^ percentile (lower whisker) and the 95^th^ percentile (upper whisker), dots represent the outliers and different letters indicate differences between the mean values of different groups.


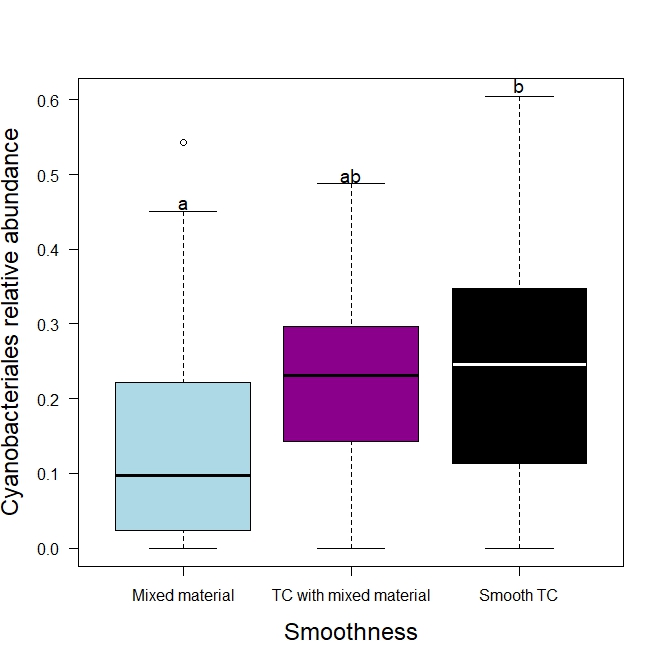


**Figure S5.** Boxplots of the relative abundances of cyanobacteriales in different levels of rock smoothness. The thick lines represent the median, boxes upper and lower limits the 25^th^ and the 75^th^ percentiles respectively, whiskers the data that go beyond the 5^th^ percentile (lower whisker) and the 95^th^ percentile (upper whisker), dots represent the outliers and different letters indicate differences between the mean values of different groups.


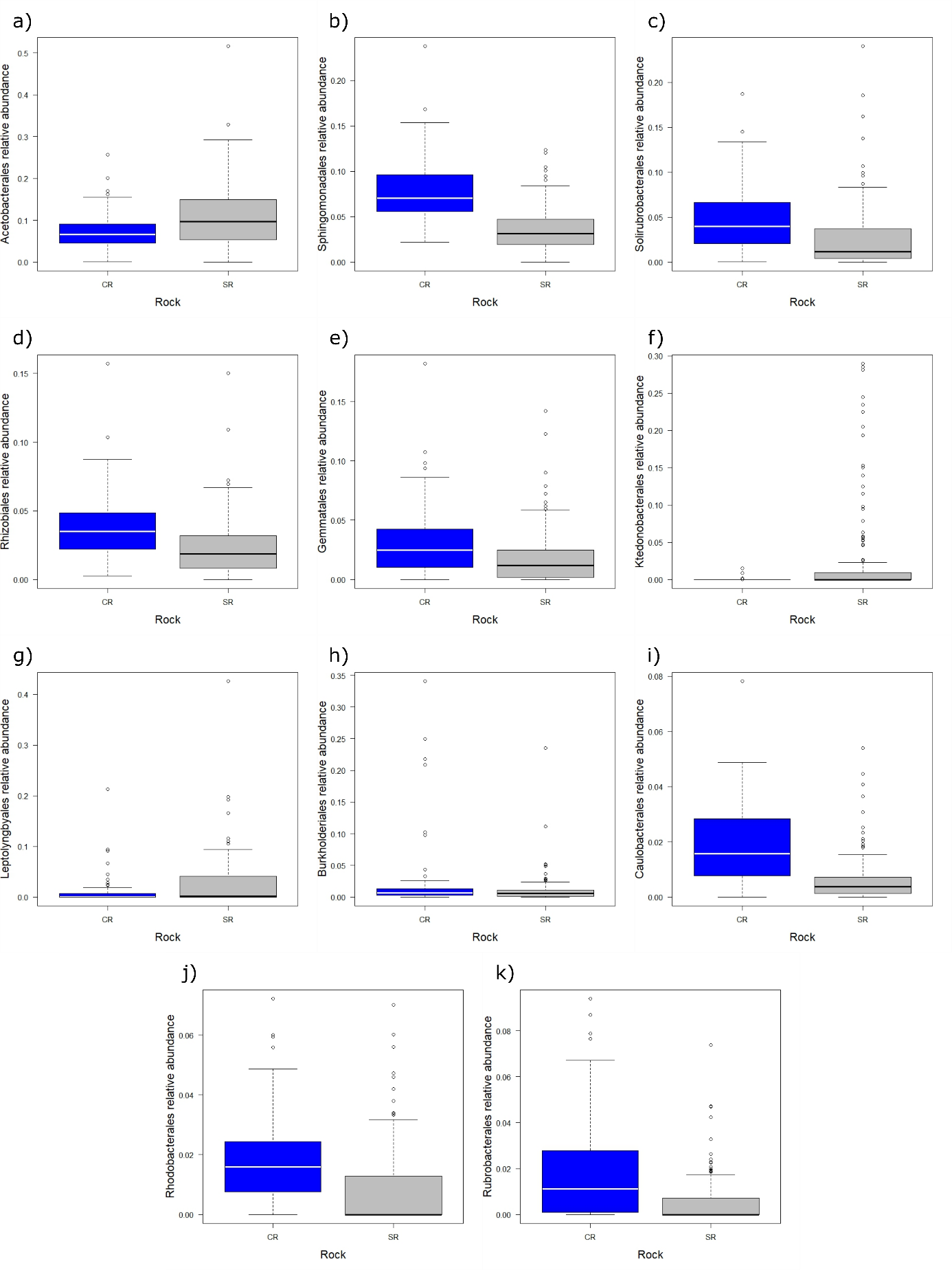


**Figure S6.** Boxplots of the relative abundances of acetobacterales (a), sphingomonadales (b), solirubrobacterales (c), rhizobiales (d), gemmatales (e), ktedonobacterales (f), leptolyngbyales (g), burkholderiales (h), caulobacterales (i), rhodobacterales (j) and rubrobacterales (k) showing differences according to the rock substrate (blue = CR (carbonatic rocks), grey = SR (siliceous rocks)). The thick lines represent the median, boxes upper and lower limits the 25^th^ and the 75^th^ percentiles respectively, whiskers the data that go beyond the 5^th^ percentile (lower whisker) and the 95^th^ percentile (upper whisker), dots represent the outliers and different letters indicate differences between the mean values of different groups.


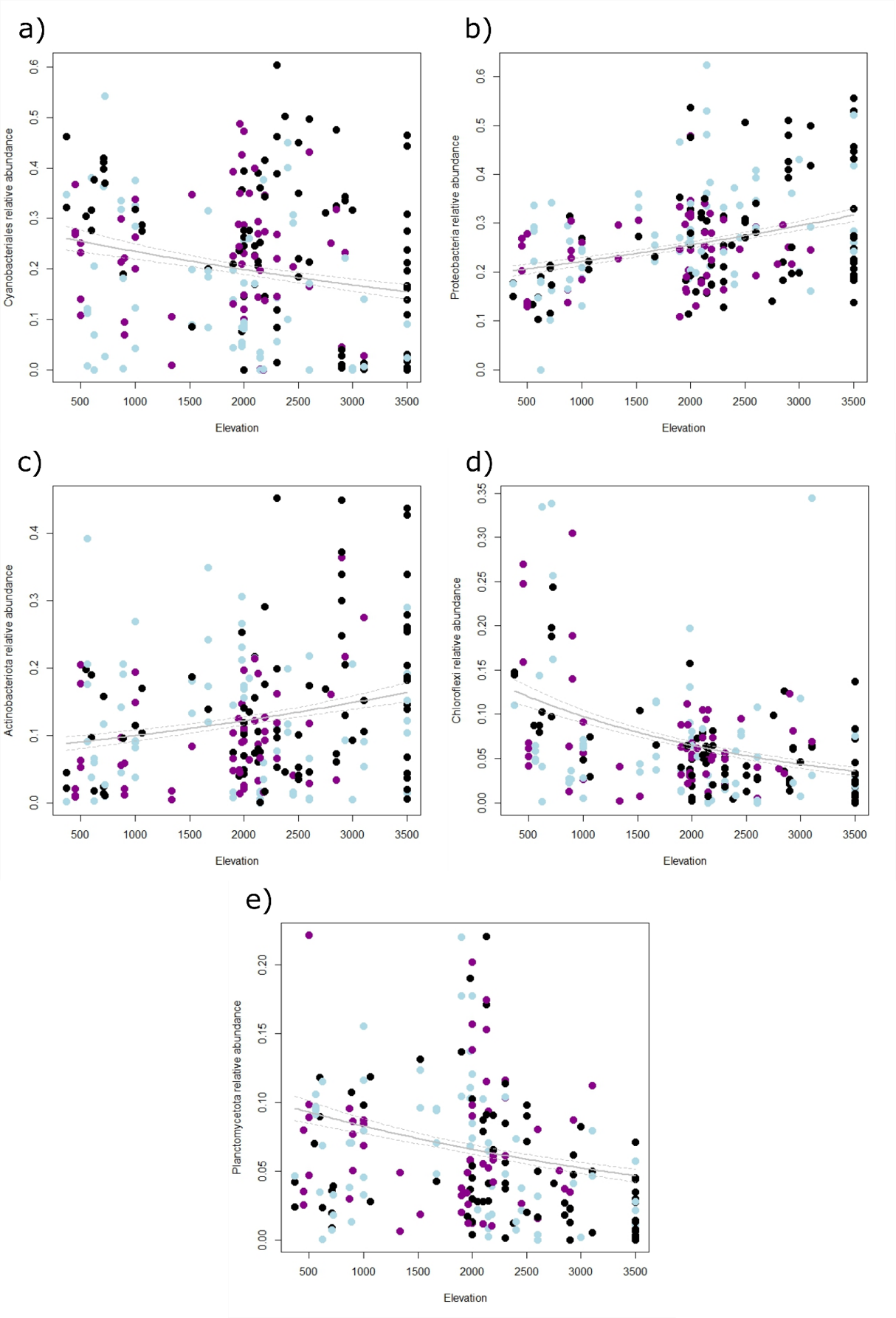


**Figure S7.** GLM plots showing the variation of cyanobacteria (a), proteobacteria (b), actinobacteriota (c), chloroflexi (d) and planctomycetota (e) with elevation. The smoothness is indicated by different colours (black = smooth tintenstrich, purple = Tintenstrich with other material, light blue = Mixed material).


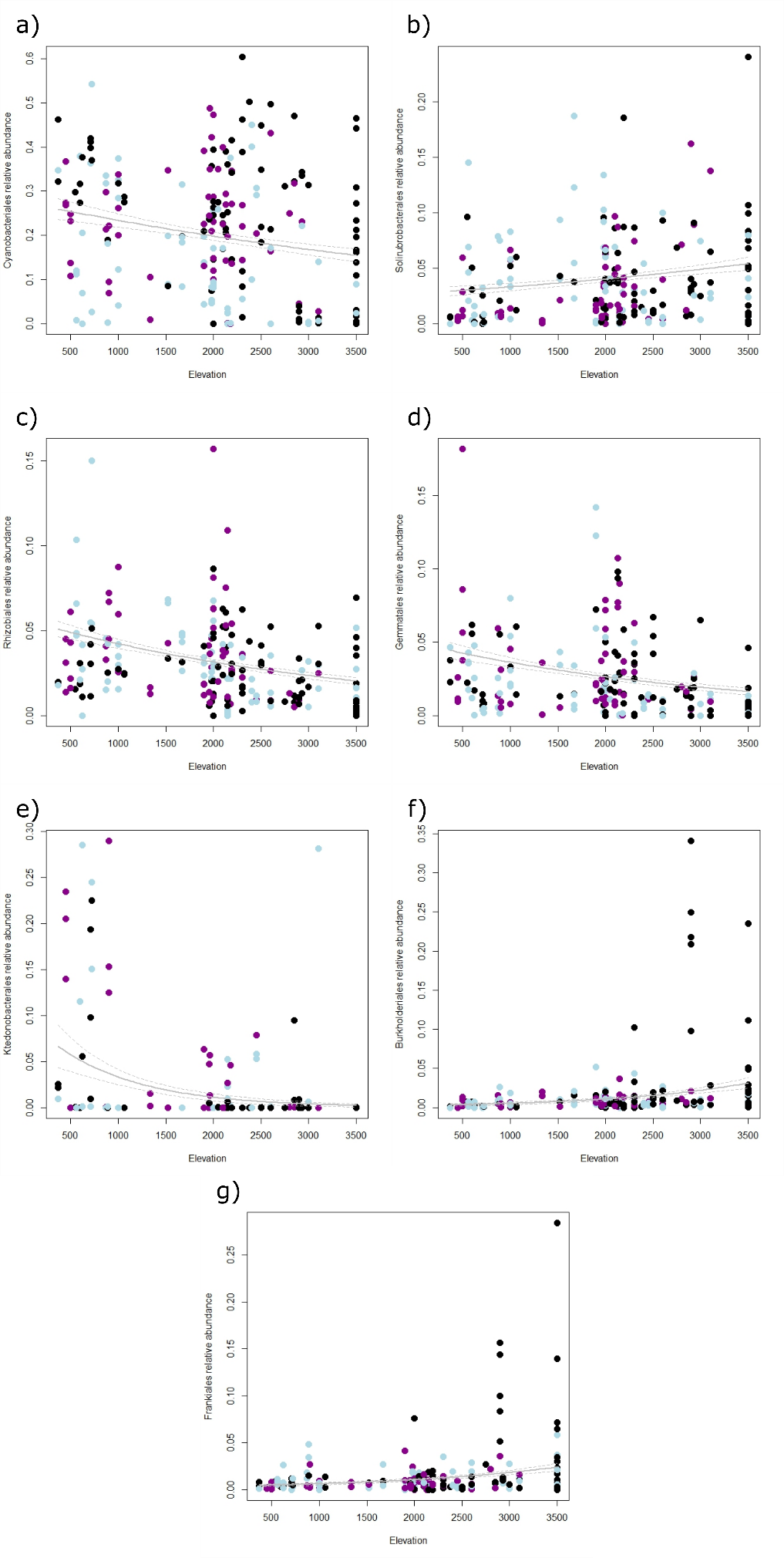


**Figure S8.** GLM (Generalized Linear Model) biplots showing the trend of Cyanobacteriales (a), Solirubrobacterales (b), Rhizobiales (c), Gemmatales (d), Ktedonobacterales (e), Burkholderiales (f) and Frankiales (g) according to elevation.


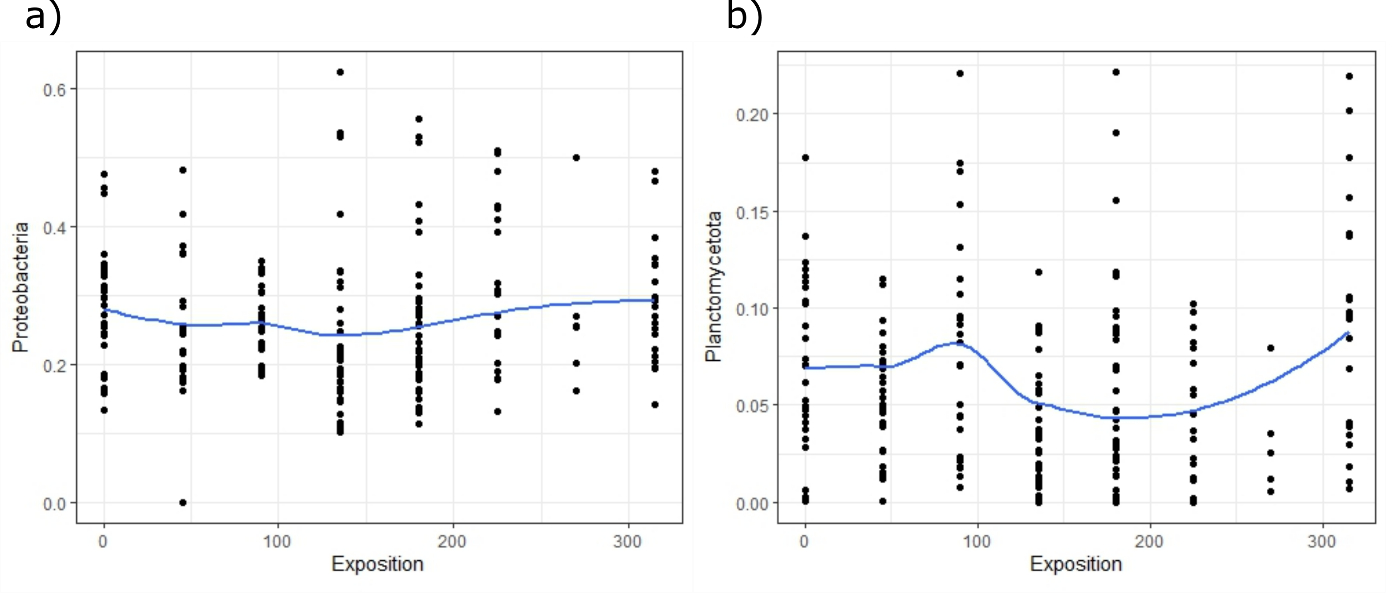


**Figure S9.** GLM (Generalized Linear Model) biplot showing the variation of proteobacteria (a) and planctomycetota (b) according to exposition.


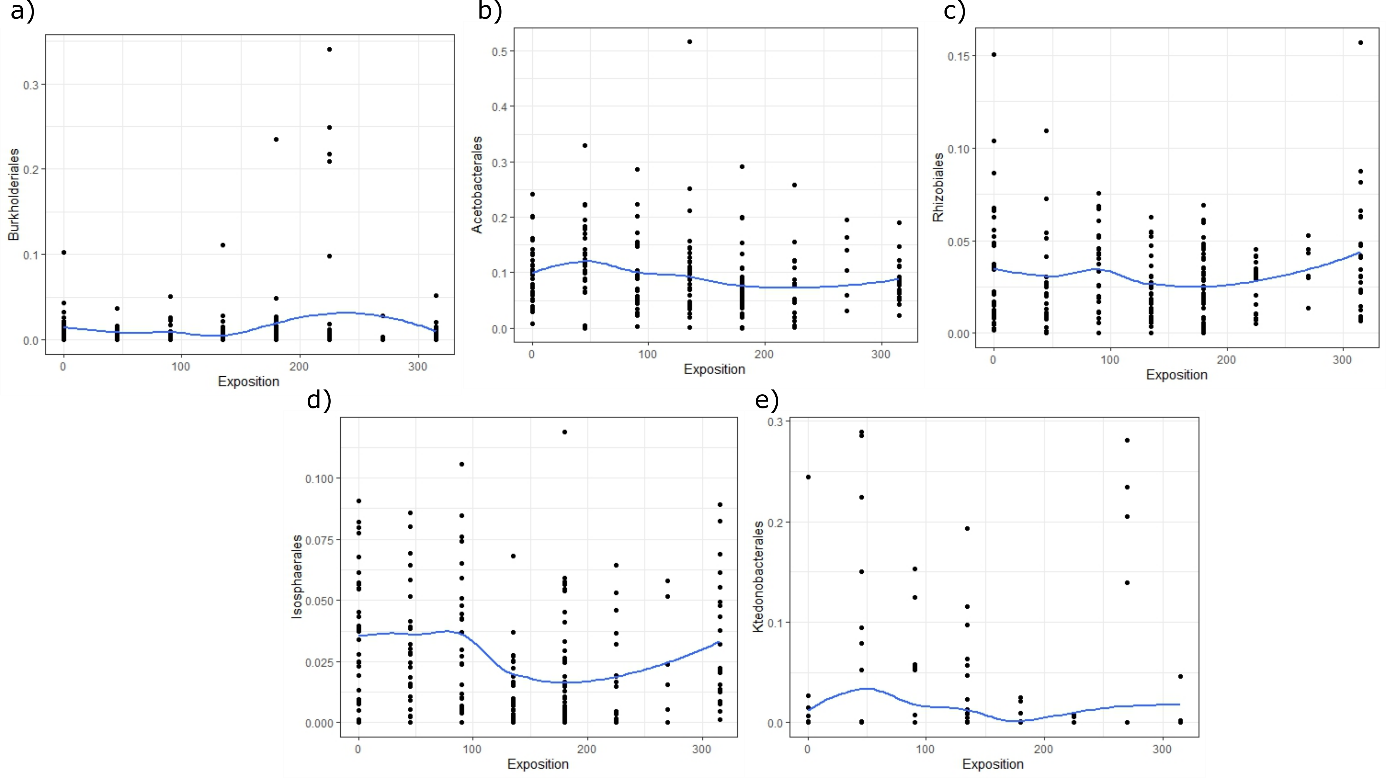


**Figure S10.** GLM biplots showing the trend of Burkholderiales (a), Acetobacterales(b), Rhizobiales (c) and Isosphaerales (d) and Ktedonobacterales (e) according to exposition. Exposition is expressed as Azimuth degrees.

**Table S1.** List of samples and their respective coordinates.
